# Biodegradation of low-density polyethylene (LDPE) sheet by microalga, *Uronema africanum* Borge

**DOI:** 10.1038/s41598-021-96315-6

**Published:** 2021-08-26

**Authors:** Elumalai Sanniyasi, Rajesh Kanna Gopal, Dinesh Kumar Gunasekar, Preethy P. Raj

**Affiliations:** 1 Department of Biotechnology, University of Madras, Guindy Campus, Chennai, 600025 India; 2 Department of Plant Biology and Plant Biotechnology, Presidency College (Autonomous), Chennai, 600005 India

**Keywords:** Biotechnology, Plant sciences, Environmental sciences, Ocean sciences

## Abstract

Plastic (polyethylene) pollution is a severe cause of deterioration of a healthy environment. For example, ingestion of plastics in the animal gut, clogging of water canals and retarded solid waste management. Many conventional methods of polyethylene degradation include UV photooxidation, thermal oxidation, incineration, chemical oxidation and landfill are being practiced. However, these methods are not feasible, costlier and not a complete solution for this global issue. Therefore, plausible, alternative solution for this issue is biodegradation. Microbes such as bacteria, fungi and algae are involved in polyethylene degradation in its natural habitat. Among them, algae were given very less importance. In our present study, a potential microalga, morphologically identified as *Uronema africanum* Borge, isolated from a waste plastic bag collected from a domestic waste dumping site in a freshwater lake. This microalga was further treated with the LDPE sheet in BBM culture medium. Based on the results obtained from light microscopy, dark field microscopy, GC–MS, FT-IR, SEM and AFM, it was concluded that the microalga has initiated degradation of LDPE sheet within 30 days of incubation. Concurrently, the configuration of corrosions, abrasions, grooves and ridges were found similar with the morphological features of the microalga. For example, the configuration of the radial disc-like attachment structure of the microalga was found corresponding to the abrasions on the surface of LDPE sheet at an average size of 20–30 µm in diameter. Whereas, the configuration of ridges and grooves were found similar with the filamentous nature of the microalga (10–15 µm width). This is a *hitherto* report on the biodegradation of LDPE sheet by the microalga *Uronema africanum* Borge.

## Introduction

Unconstrained production of plastics by packaging industries for various sectors such as food, transportation and agriculture, have led environmental issues on its disposal and thereby causing pollution. Properties of plastics such as durability, flexibility, strength, inertness and light-weight including its cost-effective production are the major advantages. However, plastics are intractable to biodegradation in the natural environment. Therefore, conventional methods of plastic degradation are being practiced. But, the major crisis of plastic accumulation due to landfills cause expose of unwanted synthetic chemicals to healthy ecosystem and ingestion of plastics in the gut of animals^1^.

The annual plastic waste accumulation rate in the ecosystem was estimated as 25,000,000 tons^2–5^ which was very high and considerably a serious environmental issue^6,7^. In fact, the plastic waste contributes about 25% of municipal waste in Australia, 20% in the US and Germany and 7.5% in the Western Europe^8^. Annual plastic disposal was estimated as 11,000,000 tons in Turkey^9^. However, in India, annual plastic waste was estimated as 5.6 million metric tons. Among the plastic usage in India, only 60% of the total plastic was collected and recycled. The four metro cities contribute about 21.2% of total plastic waste in India, viz. 7.49% of plastic waste by Delhi, 4.66% by Chennai, 4.62% by Kolkata and Mumbai with 4.43%^10^.

Commonly practiced plastic waste disposal methods are incineration, land-filling and recycling. Henceforth, these methods are unsustainable and ineffective for plastic waste management by causing further hazardous effects to the environment. Therefore, search for a potential remedial measure is necessary in the upcoming future. One such auspicious and potential method is biodegradation of recalcitrant synthetic polymer^11^. The degradation of polyethylene by microorganisms were reported in the year 1961 by Fuhs^12^ by consuming paraffin as a carbon source. Microbes including bacteria, fungi and algae have been reported in plastic degradation in the natural habitat^13^. Microbes from variety of sources such as polyethylene buried in the soil, marine water, plastic dumping sites and rhizosphere soil of mangroves are reported to be a potential source for plastic degradation.

Biodegradation of plastic waste is an economically cheaper process, more efficient than any other conventional methods of plastic waste disposal. Similarly, the secondary products of biodegradation may exhibit economic value rather than incineration and landfill^14^. Biodegradation is a complex process involves steps including biodeterioration, depolymerization and mineralization. Hence, the biological agent utilizes the synthetic polymer as a substrate for carbon source, thus the end-product would be microbial biomass.

Some issues in the biodegradation of plastic are hydrophobicity and size of the polymer molecules^6,15^. Biodegradation of high molecular weight polymers are reported in some microbes with the help of extracellular enzymes, which can degrade the main polymeric chain and results in low molecular weight products with modified mechanical properties, which can be easily deal by microbial degradation^16,17^. In the last decade, many biodegradable plastics have been introduced in the market. Since, none of them is biodegradable in landfills. Therefore, these biodegradable plastics have not attained widespread attention globally^18^. And now, it an urgent need to promote a microbial plastic degradation process.

Even though, enormous studies have been demonstrated on the biodegradation of plastics by microbes, none of them applied practically to solve this issue. Hence, efficient screening of microorganisms for biodegradation of plastics is the need of the hour^19^. Carbon and hydrogen are the backbone of polyethylene and thus, it is resistant to biodegradation. Therefore, prior treatment is required before biodegradation, which are sunlight and oxygen treatment induces brittleness and loss of tensile strength which makes the plastic into smaller pieces^20^. Biodegradation of polyethylene by bacteria and fungi have been reported enormously, whereas algae on polyethylene degradation has attained a very meager attention^21–23^. In the context of biodegradation of polyethylene, microalgal growth on the surface of polyethylene to be given much importance^7^.

Microalgae are photoautotrophic microbes reported to colonize on the surface of polyethylene sheets submerged in wastewater^21,24^. Fritsch, reported that algae including chaetophorales, diatoms (Bacillariophyceae) and blue-green algae (Cyanophyceae) synthesize EPS (Extracellular Polymeric Substance) to adhere the substratum^25^. Microalgae biologically synthesize EPS which are the primary colonizers on wall, rocks and other substrata and results in biodeterioration of the substrata^26^. Surface charge, hydrophobicity and electrostatic forces are the unique properties of EPS which binds to the substrata^27^.

Therefore, an attempt has been made in our present study to identify a potential microalga for biodegradation of low-density polyethylene (LDPE).

## Results

### Collection of samples

**Figure 1:**
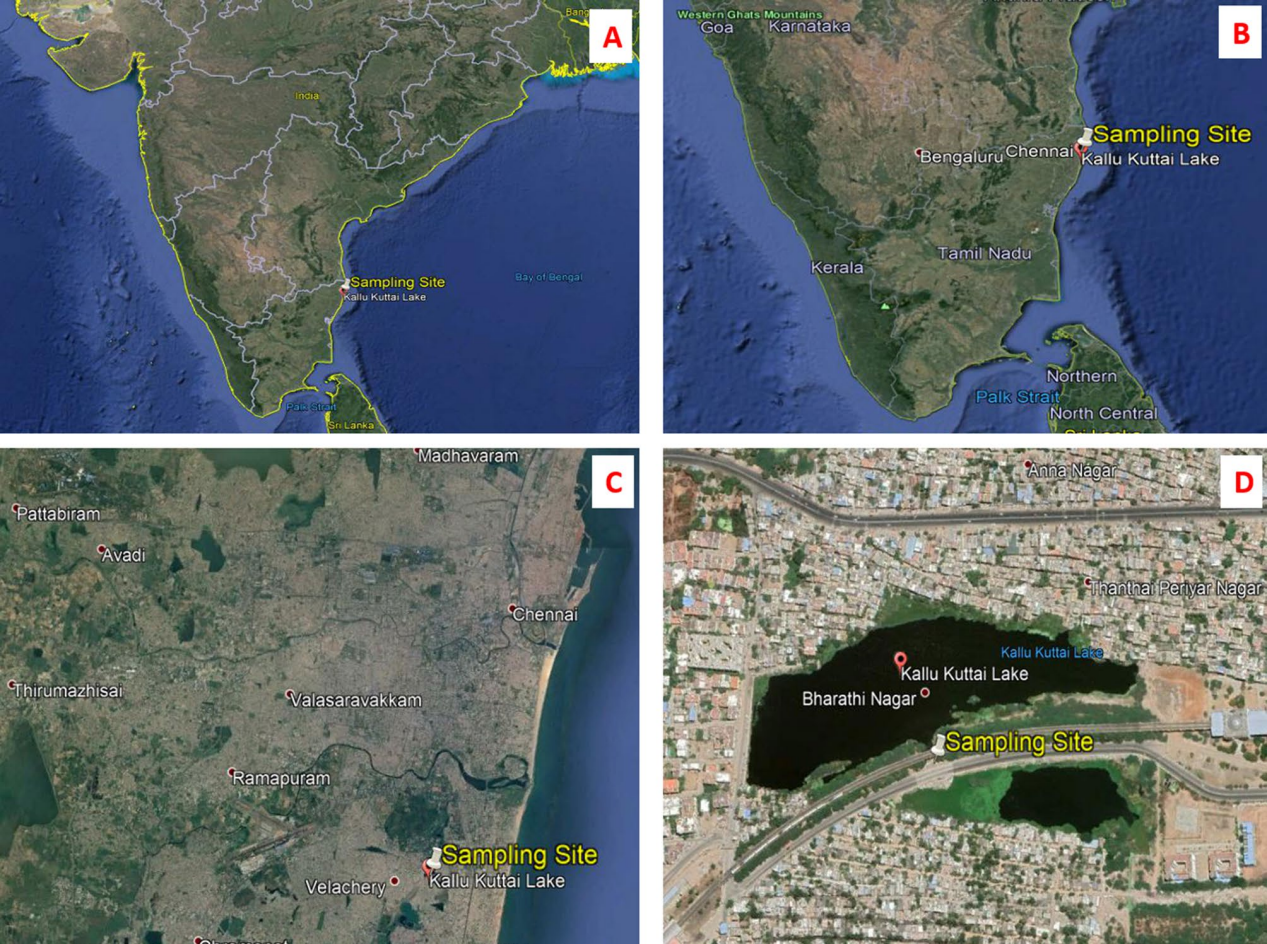
(**A**) Topographical view of the Indian sub-continent showing all states and its borders, where the sample were taken from the state of Tamil Nadu (TN) situated down-south of India; (**B**) The image showing topographical view of the state TN, showing the sampling site in the city Chennai, present top North East of TN; (**C**) Figure showing the Chennai city, where the sampling site is located nearby Velachery town; (**D**) This image showing the exact sampling location (Longitude: 12°58′41.60″ North and Latitude: 80°14′08.43″ East) of Kallukuttai Lake, nearby Taramani Railway Station, Chennai, Tamil Nadu, India showing rapid urbanization on all the borders of the lake (Google Earth Pro 7.3.3.7699).

Figure 1 showing topo geographical view of the sample collection site, Kallukuttai Lake, located near Taramani Railway Station, Chennai City, Tamil Nadu, India with longitude and latitude positions 12°58′41.70″ North and 80°14′08.57″ East respectively. The site was chosen based on the dumping of domestic wastes along the freshwater bodies due to rapid urbanization.

**Figure 2:**
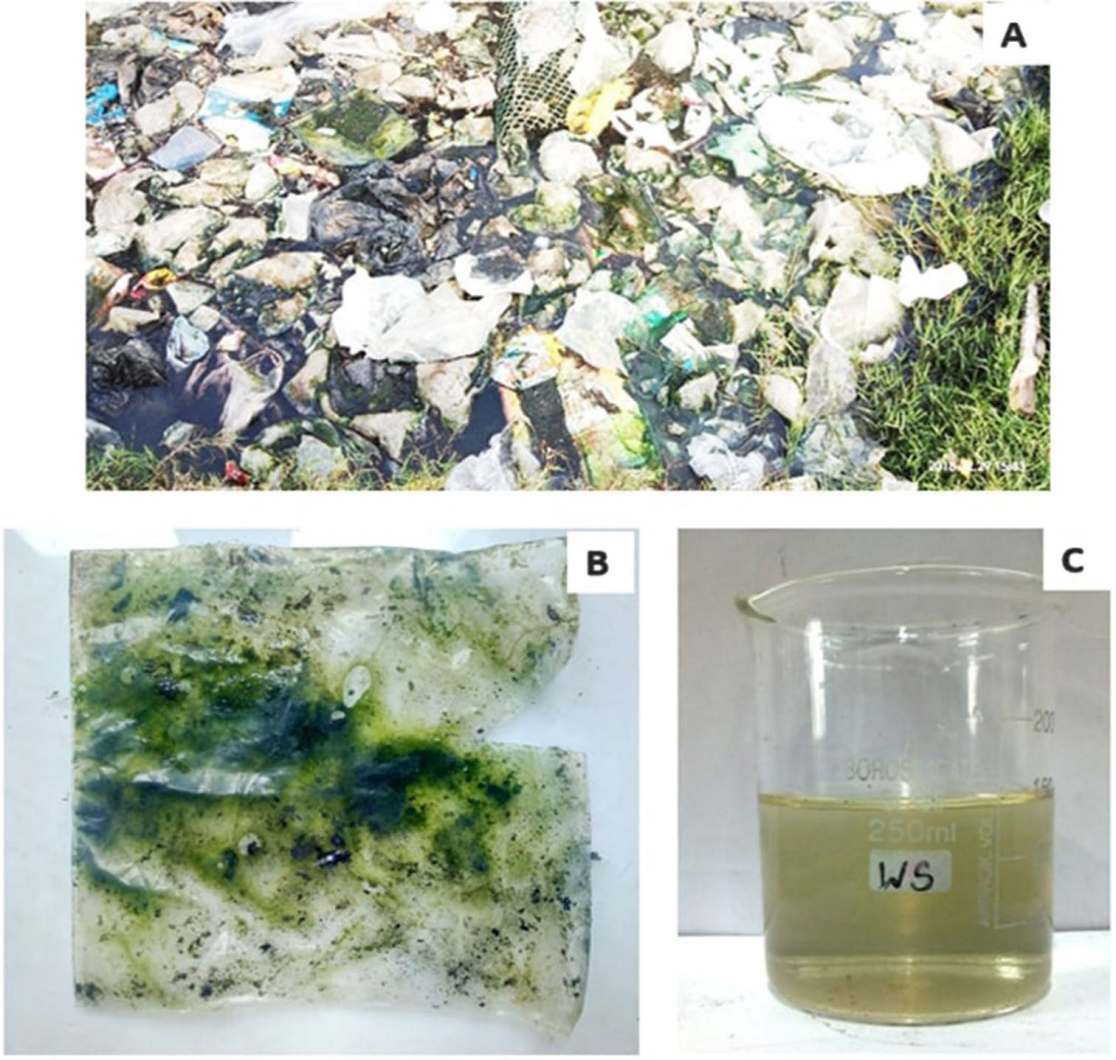
(**A**) The sampling site showing dumped waste plastic carry bags and other wastes; (**B**) Dumped waste plastic sheet found colonized by microalgae; (**C**) Water sample collected from the sampling site.

**Figure 3:**
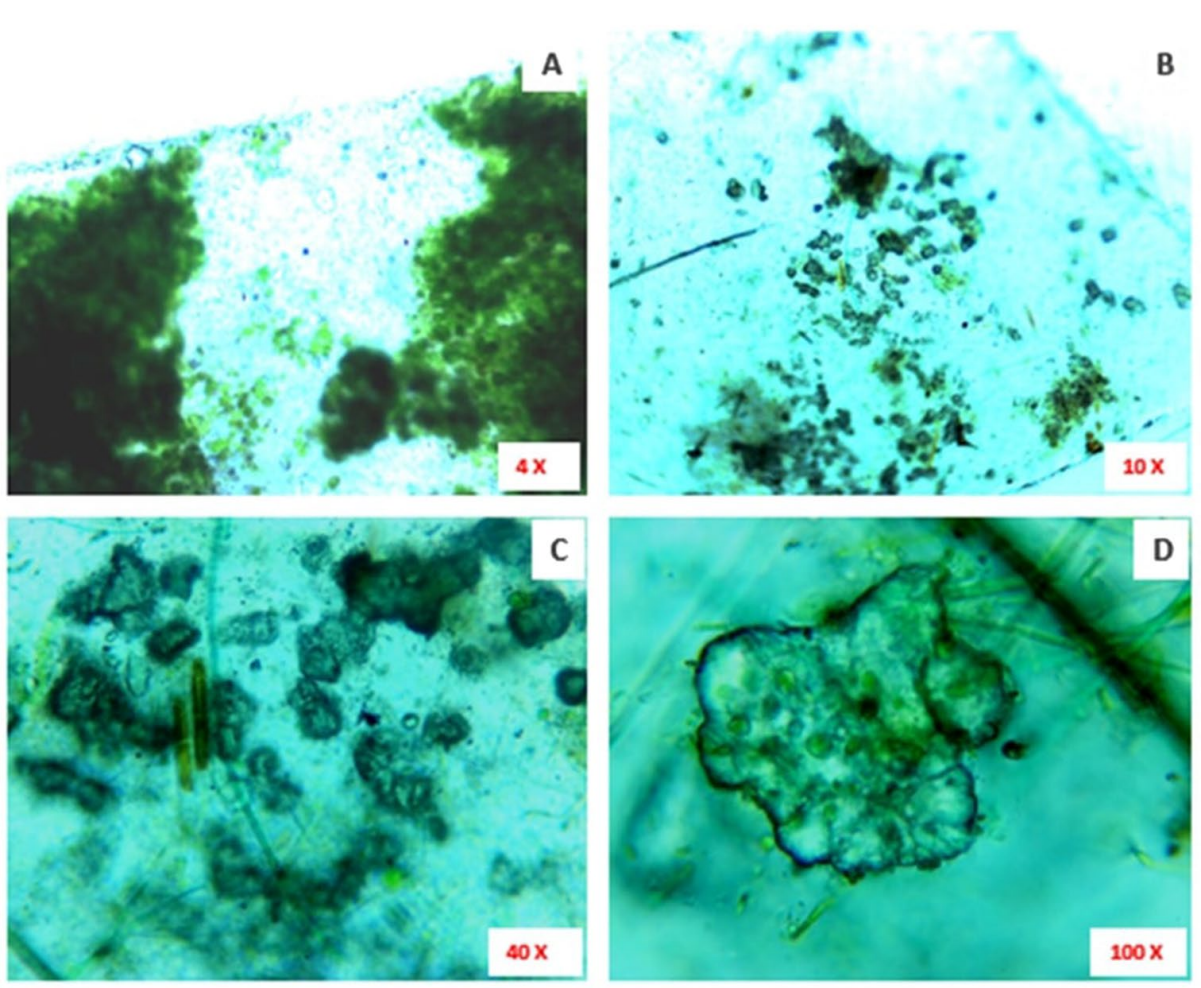
(**A**) The microscopic image (× 10 magnification) showing the colonization of green photosynthetic microalgae on the surface of the collected plastic sheet from the sampling site; (**B**) The × 10 magnification of collected waste plastic sheet showing erosions on its surface; (**C**) The × 40 magnification also represents the erosions on the surface of the collected plastic sheet; (**D**) The × 100 magnification showing a part of eroded surface on the collected plastic sheet.

A lump of plastic carry bag waste was found dumped along the borders of freshwater bodies at Kallukuttai Lake, near Taramani Railway Station, Chennai (Fig. 2A). Where, we have collected a polyethylene carry bag sample colonized by green photosynthetic microalgae and water sample (Fig. 2B,C). The light microscopical view of collected polyethylene sample shown colonized by microalgae (Fig. 3A) and abrasions on the surface of the polyethylene sheet at different magnifications (Fig. 3B–D).

### Isolation of microalga colonized on LDPE sheet

**Figure 4:**
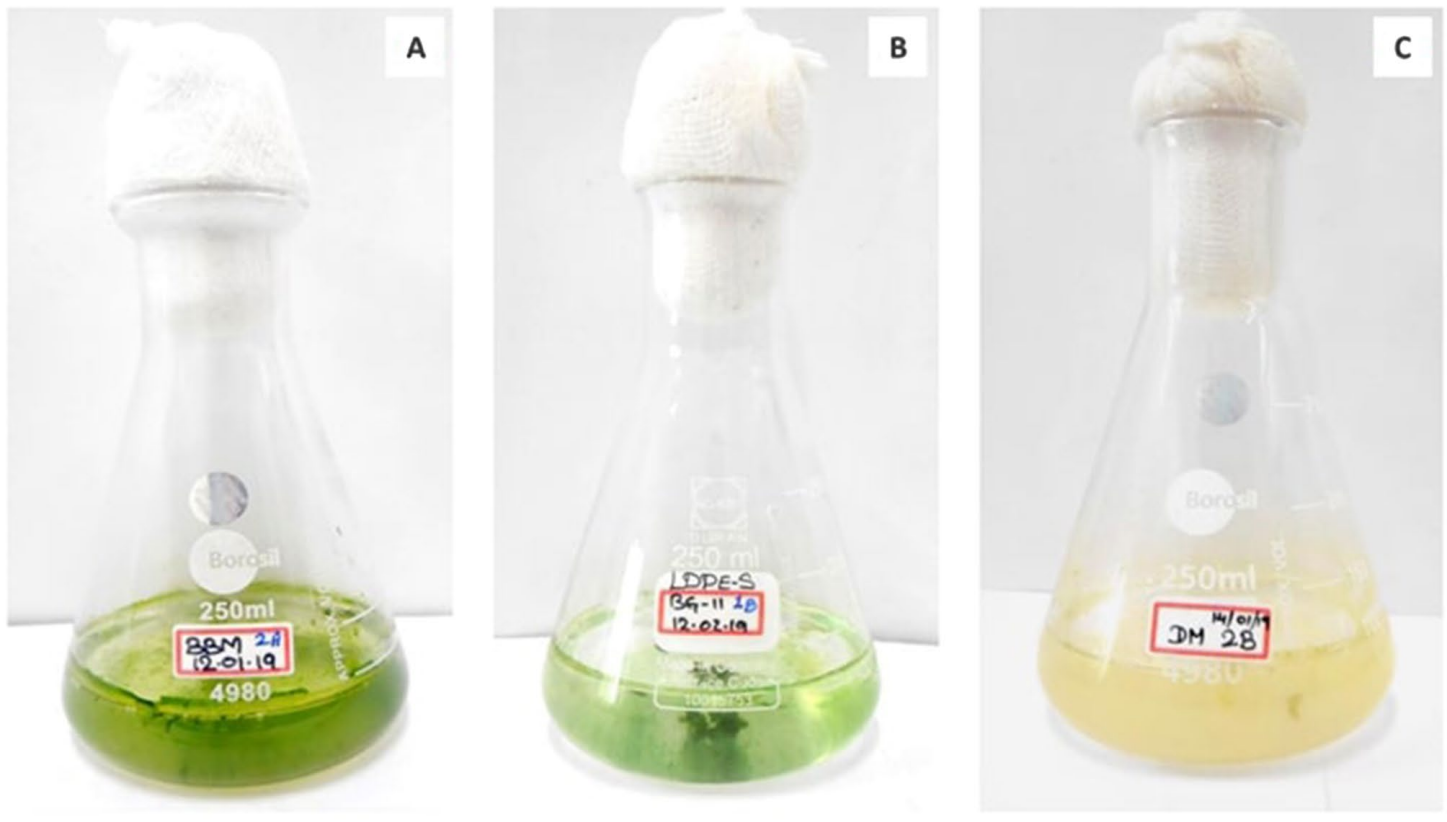
The three conical flasks showing different culture medium to target different microalgae colonize on LDPE sheet, (**A**) BBM culture medium for Chlorophyceae; (**B**) BG-11 for Cyanophyceae and (**C**) DM for Bacillariophyceae.

**Figure 5:**
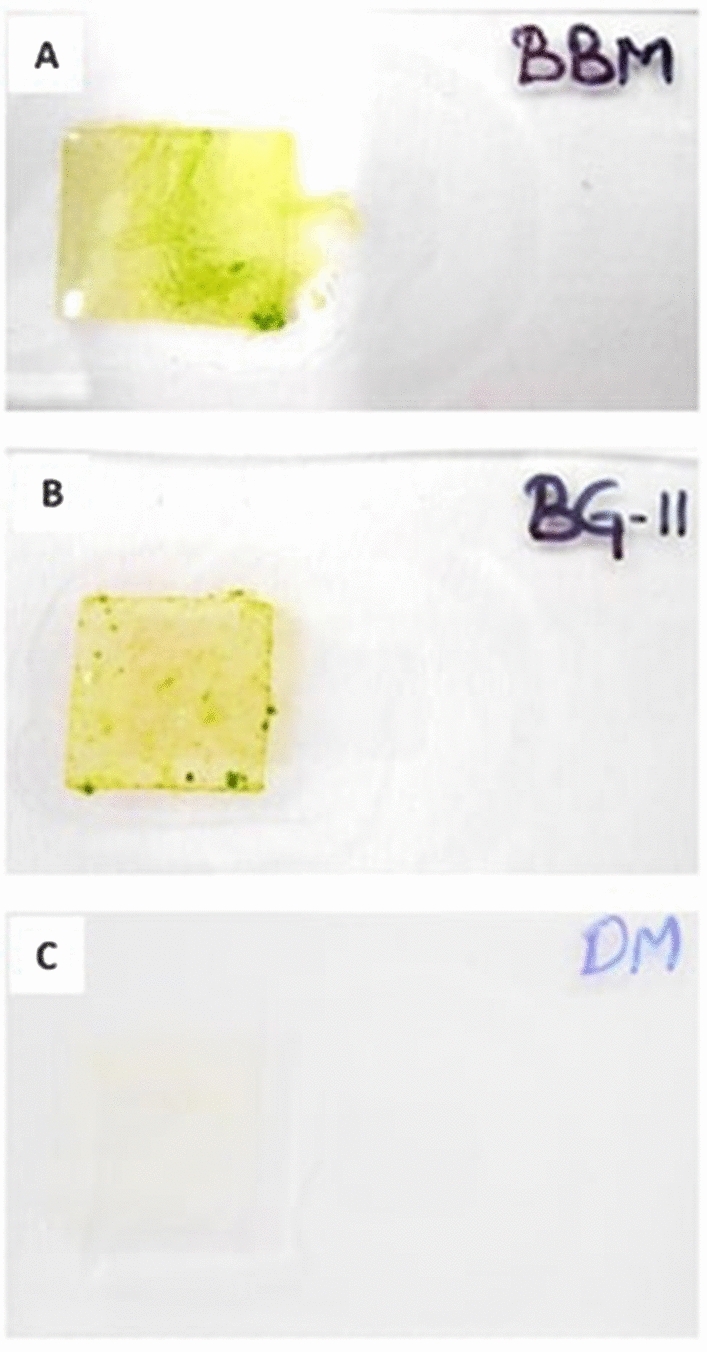
The LDPE sheets colonized by microalgae; (**A**) LDPE sheet supplemented in BBM culture medium shown colonized by green mass of algae; (**B**) LDPE sheet supplemented in BG-11 culture medium shown colonized by algae, but not like as in BBM culture medium and (**C**) LDPE sheet supplemented in DM culture medium shown very least colonization.

**Figure 6:**
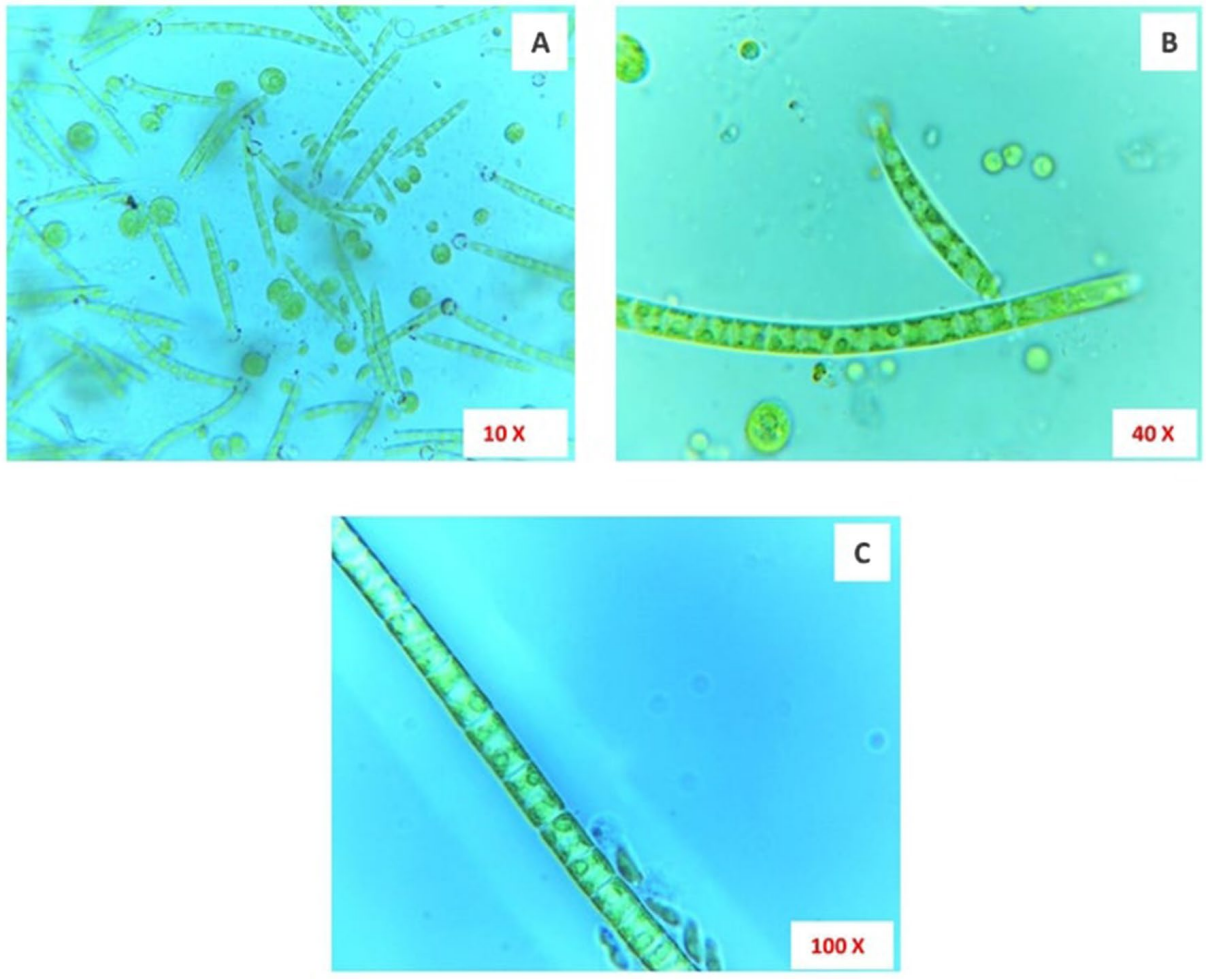
Microscopical images of isolated green algae colonized on the LDPE sheet. (**A**) × 10 magnification; (**B**) × 40 magnification and (**C**) × 100 magnification.

Three different groups of microalgae were targeted for the biodegradation of LDPE sheet (Fig. 4). As a result, the LDPE sheet was colonized by green algae (Chlorophyceae) supplemented in BBM culture medium, whereas other two culture medium have shown very low colonization (Fig. 5). Therefore, the highly colonized green microalga was isolated and maintained in the Algal culture room for further processes (Fig. 6).

### Morphological identification of selected microalga

**Figure 7:**
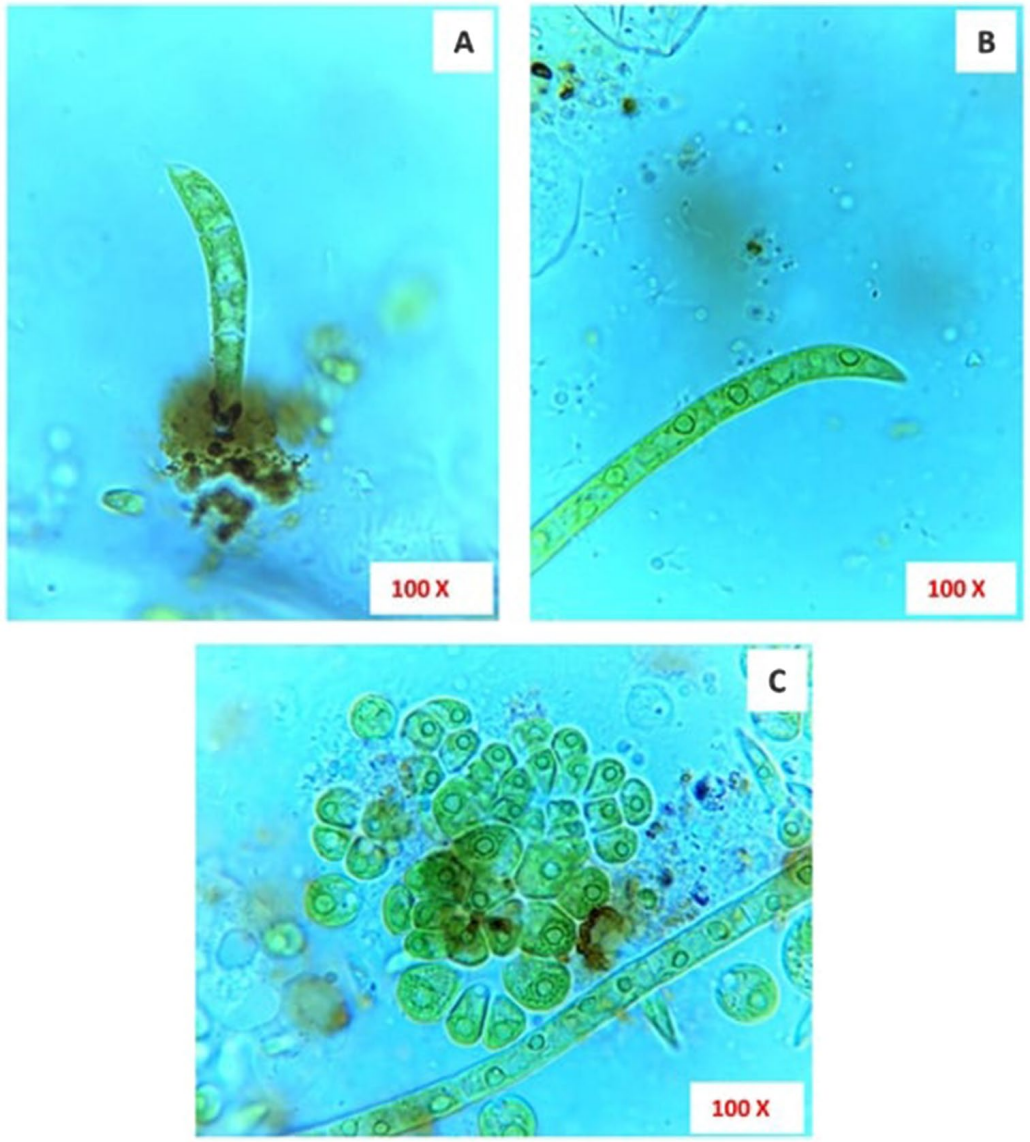
Microscopical images of green microalga colonized on LDPE sheet, *Uronema africanum* Borge, (**A**) Four celled-stage, showing apical cell slightly curved like sickle and a radial disc like attachment on the LDPE sheet; (**B**) An elongated filament showing similar sickle like apical cell; (**C**) Two different stages of *Uronema africanum* Borge with ‘C’ shaped chloroplast.

The isolated microalga colonized on LDPE sheet was grown as a filament with uninucleate cells and thus it was classified under the Order Ulotrichales and Family Ulotrichaceae^35^. The cells in the filaments are prominently free and not grouped or clustered with a pointed apical cell. Thus, the genus of the isolated microalga was identified as *Uronema*. The cells are scarcely constricted at the septum, terminal cell much curved and pointed like a sickle. Chloroplast looks ‘C’ shaped with only one pyrenoid. The basal cell elongated and shorter than other intercalary cells and enlarged into an attaching disc (Fig. 7). Based on these characteristic features, the isolated green microalga was identified as *Uronema africanum* Borge^28^. This species has been recorded in Africa, Asia and Europe and differs from *Uronema confervicolum* by having curved and pointed apical cell. It was reported in Rangoon, Burma by attaching to other algae and plants as epiphytes^29^.

Based on the modern phylogenetic system of classification, the systematic position of the isolated microalgal species *U. africanum* Borge was retrieved from algaebase database^30^.

### Systematic classification

Empire: Eukaryota
Kingdom: Plantae
Subkingdom: Viridiplantae
Infrakingdom: Chlorophyta infrakingdom
Phylum: Chlorophyta
Subphylum: Chlorophytina
Class: Chlorophyceae
Order: Chaetophorales
Family: Uronemataceae
Genus: *Uronema*
Species: *africanum* Borge

### Biodegradation of LDPE sheet by selected microalga

**Figure 8:**
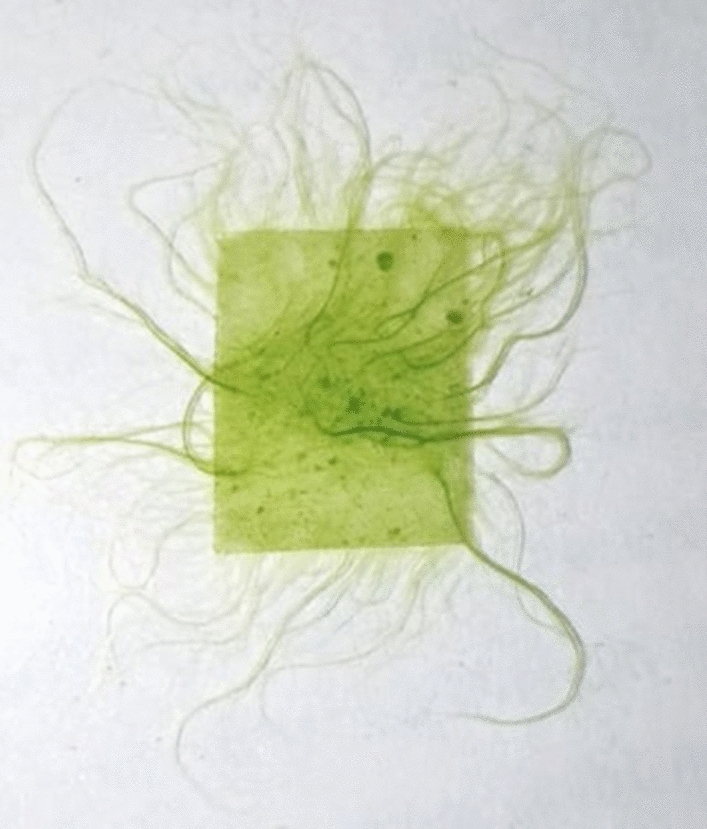
Microalga treated LDPE sheet (1 cm^2^) showing green hair-like structures protruding from the LDPE sheet.

The isolated, pure microalga *Uronema africanum* Borge was then subjected to treat with LDPE sheet in BBM culture medium. After 30 days of incubation, the LDPE sheet was fully colonized by microalga and visualized like green hair-like structures protrude from the LDPE sheet (Fig. 8).

### Optical microscopy on biodegradation of LDPE sheet

**Figure 9:**
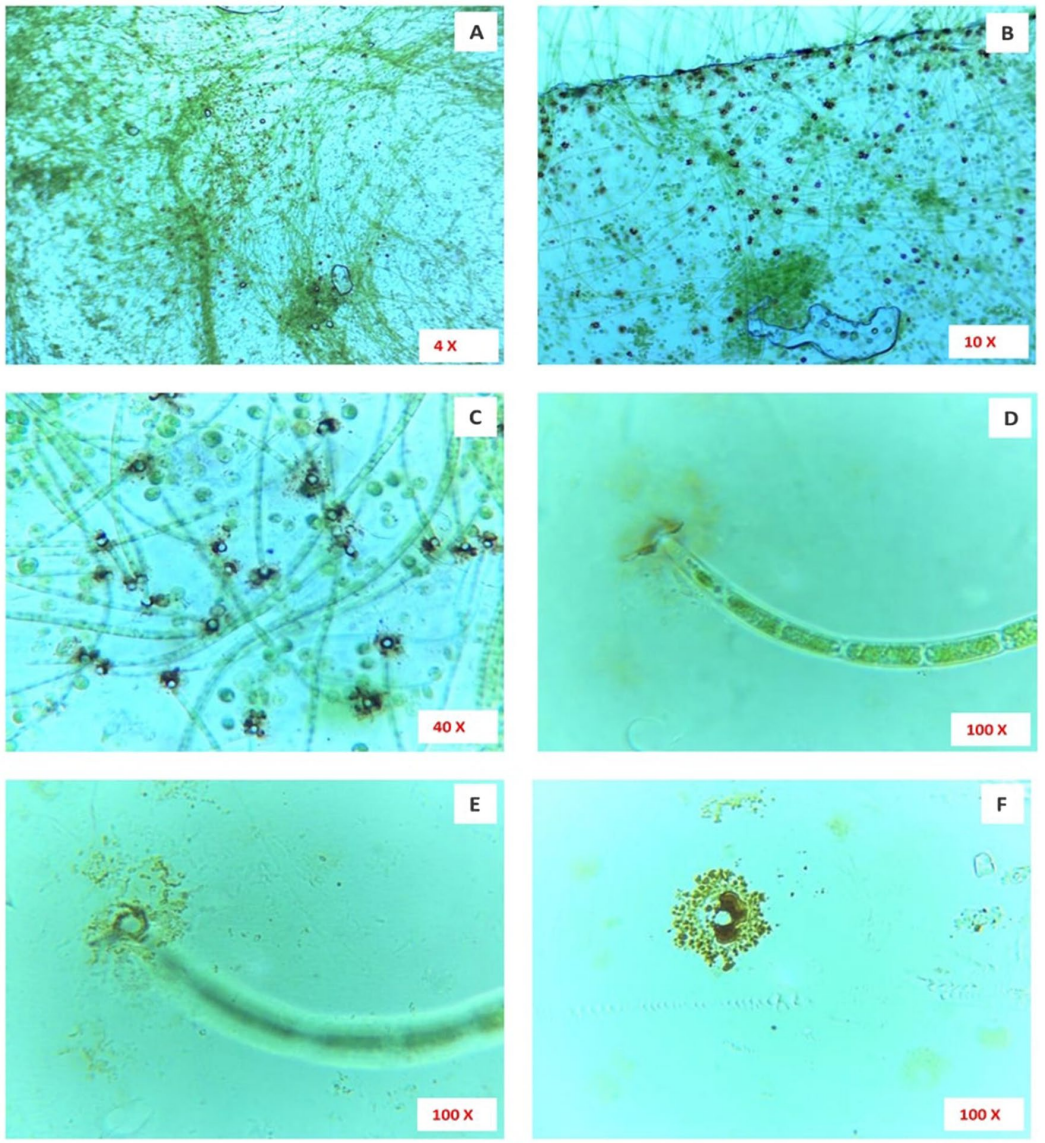
The microscopic images showing colonization of microalga *Uronema africanum* on the surface of LDPE sheet. (**A**) The × 4 magnification image showing small dot-like structures and hair-like structures on the LDPE sheet; (**B**) In × 10 magnification, the hair-like structures are nothing but the filamentous structures of the microalga *Uronema africanum* protrude from the black dots; (**C**) The × 40 magnification of microalga treated LDPE sheet; (**D**) The × 100 magnification illustrates a single filament found attached to the LDPE sheet by forming a radial disc-like structure by the basal cell; (E) The radial disc is clearly focused in × 100 magnification; (**F**) The radial disc without microalgal filament.

**Figure 10:**
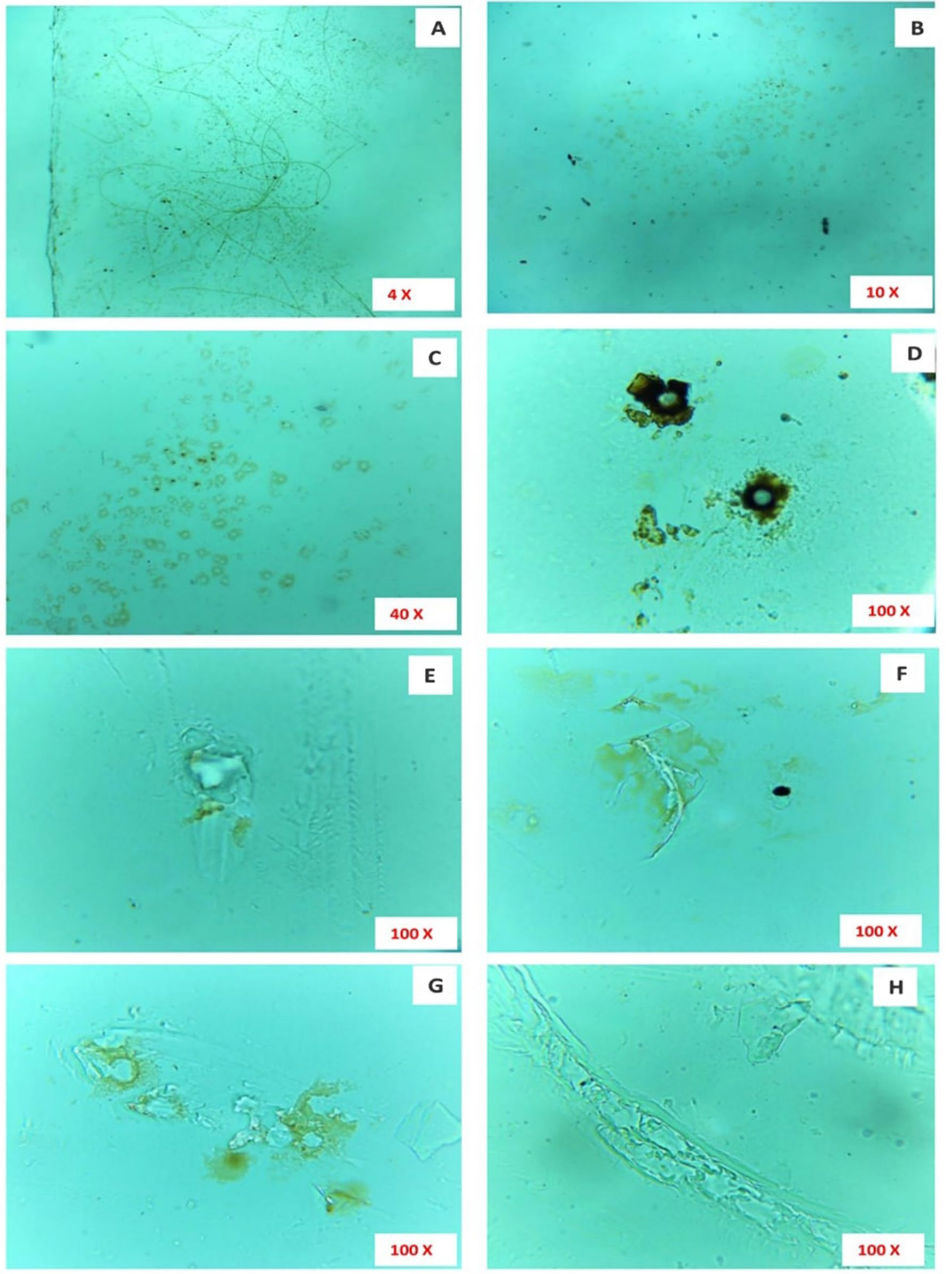
Microscopically magnified, washed LDPE sheet. (**A**) The × 4 magnification of washed LDPE sheet showing hair-like structures still present on the LDPE sheet; (**B**,**C**) × 10 magnification of the radial disc, still present after washing; (**D**) A × 100 magnification of radial disc after washing, with absence of microalgal filament; (**E**–**H**) × 100 microscopical images clearly indicate erosions, abrasions and grooves and ridges on the surface of the LDPE sheet.

**Figure 11:**
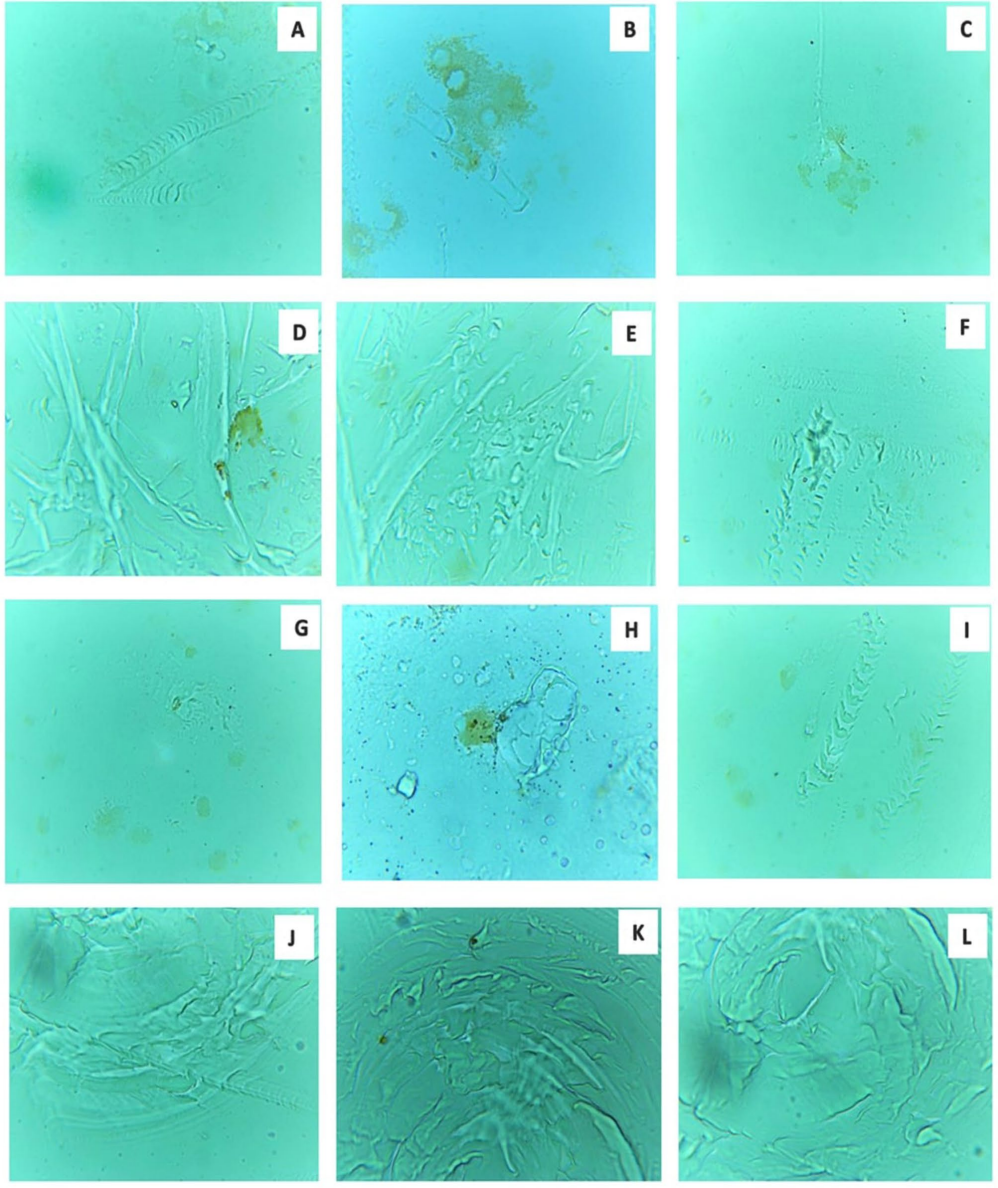
(**A**–**L**) Showing degraded surface of LDPE sheet by the microalga *U. africanum* (× 100 magnification) with erosions, abrasion and ridges and grooves on LDPE sheet.

The microscopical images of microalga (*Uronema africanum*) treated LDPE sheet, with different range of magnifications showing, microalgal colonization on the surface of the LDPE sheet by forming a radial disc on its surface (Fig. 9). Hence, the filaments are found attached to the LDPE sheet at an angle of 90° and perpendicular to the sheet. Similarly, the washed LDPE sheets showing the presence of some microalgal filament due to strong attachment on the LDPE sheet by the radial disc. The radial disc like structures are still present on the surface of LDPE sheet and some washed regions showing erosions, abrasions, grooves and ridges (Fig. 10). The erosion was formed due to the radial disc formation, whereas grooves and ridges followed by abrasions was formed by the filaments of the microalga (Fig. 11).

### Dark field microscopy on biodegradation of LDPE sheet

**Figure 12:**
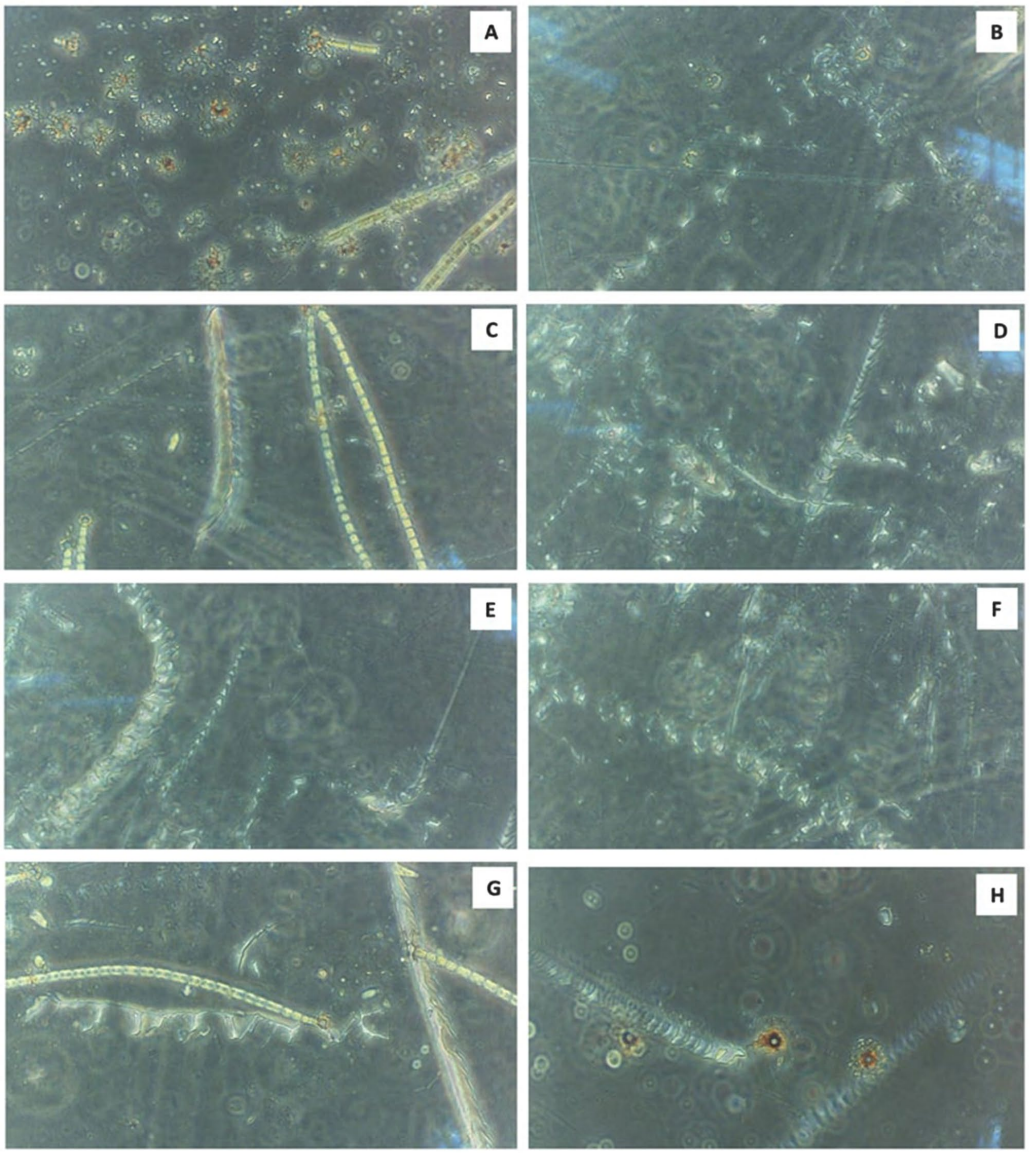
(**A**–**H**) The × 40 magnification of dark field microscopic images clearly indicates the presence of erosions on LDPE sheet due to the formation of radial disc by microalga followed by abrasions, ridges and grooves formed by the filaments of microalga *U. africanum*.

The dark field microscopical study clearly illustrates that the erosions were formed by the radial discs, whereas abrasions with ridges and grooves were formed by the filaments of the microalga *U. africanum* (Fig. 12). Furthermore, the configuration of the groove formed by the microalga correspondingly similar with the filamentous structure of the microalga *U. africanum*.

### Gas chromatography and mass spectrometric analysis of control and microalga treated LDPE sample

**Figure 13:**
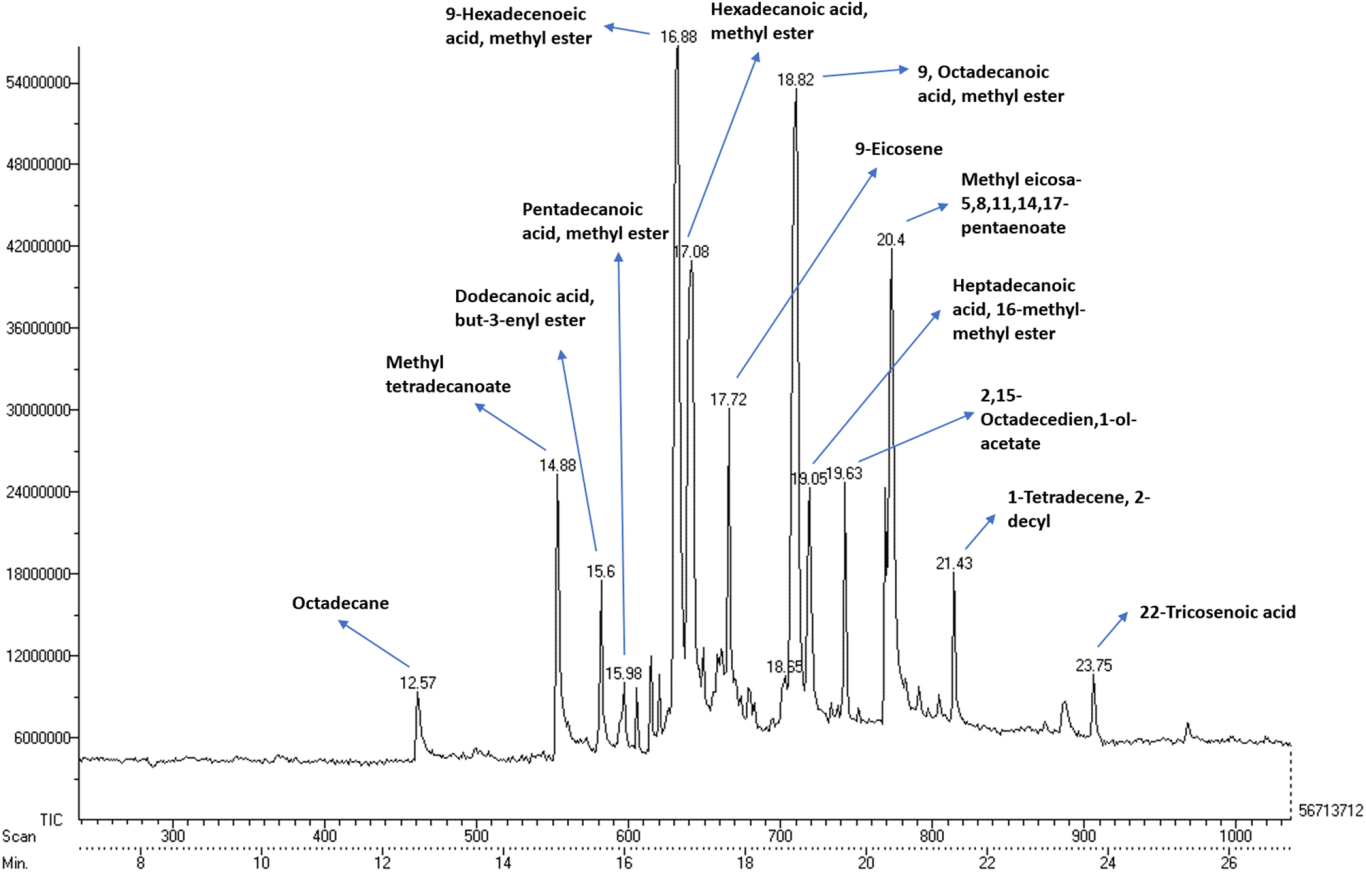
Gas chromatography spectrum of control sample (without LDPE sheet).

**Figure 14:**
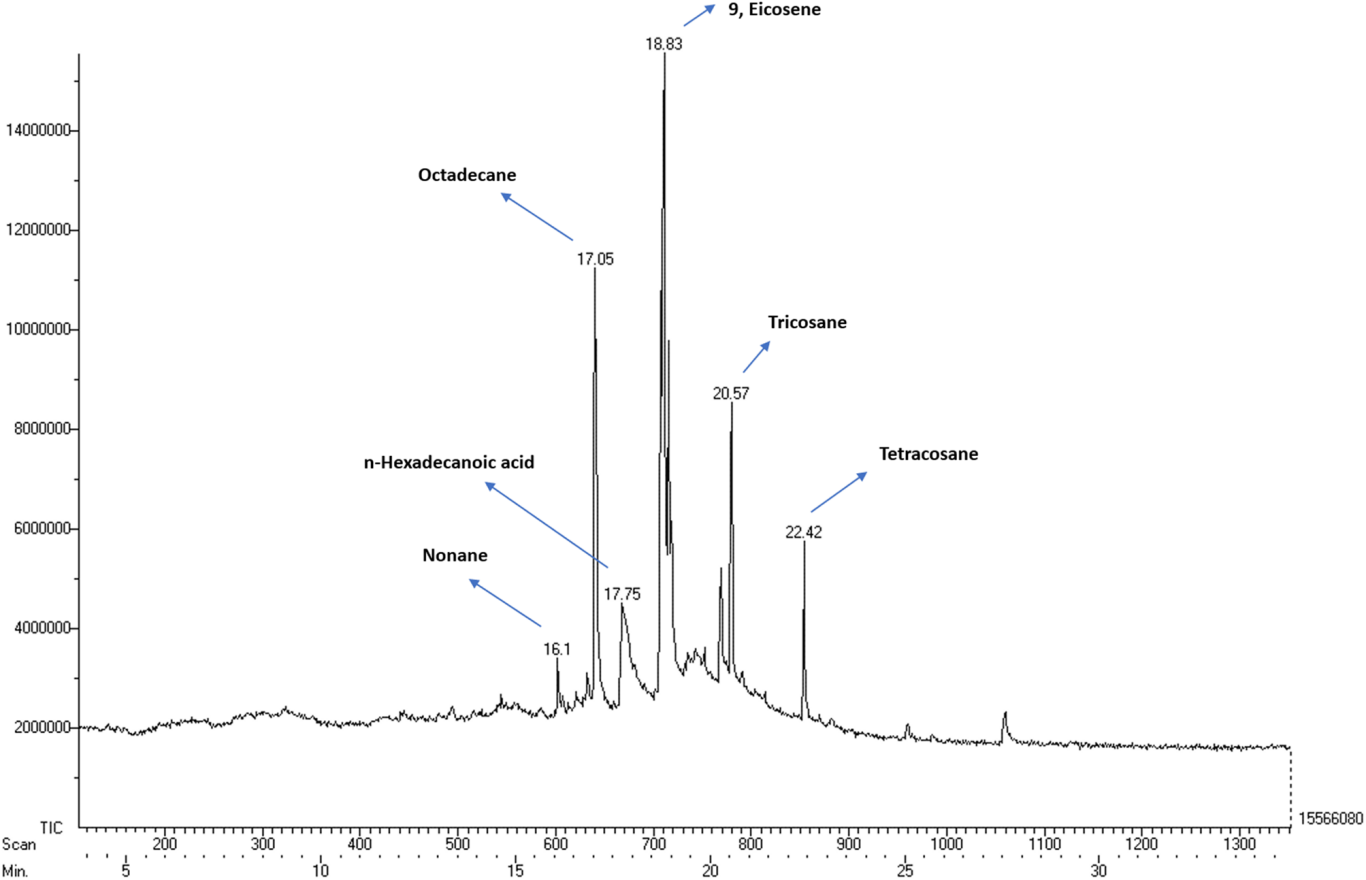
Gas chromatography spectrum of microalga treated sample (with LDPE sheet).

Based on the GC–MS analysis, there is a huge difference in the fatty acids, comparatively between the supernatants of both control (*U. africanum* culture without LDPE sheet), and sample (*U. africanum* with LDPE sheet). Fatty acids such as Octadecane (C_18_H_38_), Methyl tetradecanoate (C_15_H_30_O_2_), Dodecanoic acid, but-3-enyl ester (C_18_H_34_O_2_), Pentadecanoic acid, methyl ester (C_16_H_32_O_2_), 9-Hexadecanoic acid, methyl ester (C_17_H_32_O_2_), Hexadecanoic acid, methyl ester (C_17_H_32_O_2_), Octadecanoic acid, methyl ester (C_19_H_38_O_2_), 9-Eicosene (C_20_H_40_), Methyl eicosa 5,8,11,17-pentaenoate (C_21_H_32_O_2_), Heptadecanoic acid, 16-methyl-methyl ester (C_19_H_38_O_2_), 2,15-Octadecedien, 1-ol-acetate (C_20_H_36_O_2_), 1-Tetradecene, 2-decyl (C_14_H_28_) and 22-Tricosenoic acid (C_23_H_44_O_2_) were found in the control (Fig. 13). Whereas, Nonane (C_9_H_20_), n-Hexadecanoic acid (C_16_H_32_O_2_), Octadecane (C_18_H_38_), 9-Eicosene (C_20_H_40_), Tricosane (C_23_H_48_) and Tetracosane (C_24_H_40_) were found obtained in the sample (*U. africanum* with LDPE sheet) (Fig. 14).

Comparatively, the control (*U. africanum* culture without LDPE sheet) consists of fatty acid methyl esters. However, the microalgae treated sample (*U. africanum* with LDPE sheet) consists of large number of hydrocarbons. Therefore, it is considered that the generation of hydrocarbons might be due to the biodegradation of LDPE sheet by the respective microalga *Uronema africanum*.

### Fourier transform infrared spectroscopy (FT-IR) analysis of microalga treated LDPE sheet

**Figure 15:**
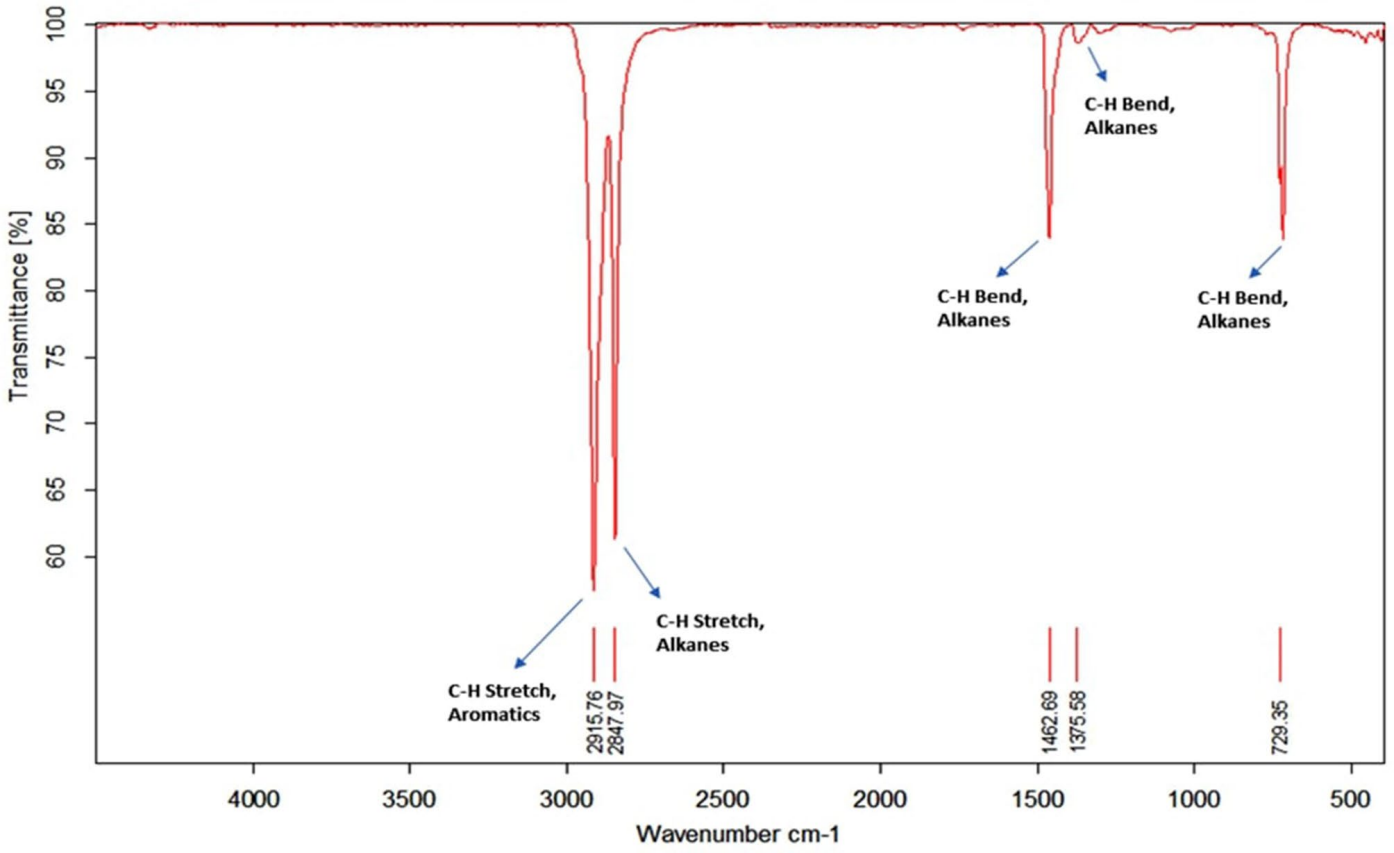
FT-IR spectrometry results of control LDPE sheet.

**Figure 16:**
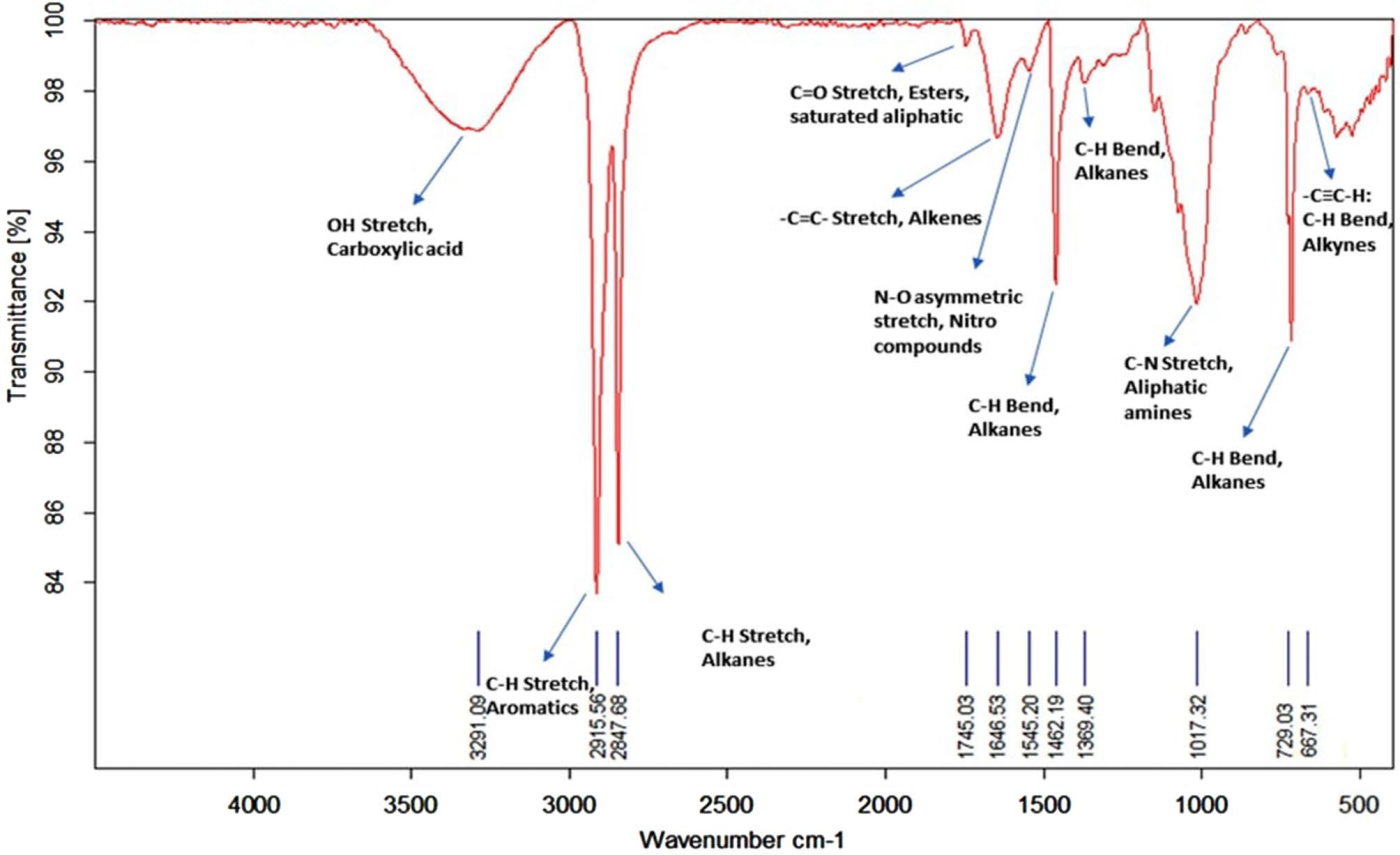
FT-IR spectrometry results of microalga (*Uronema africanum*) treated LDPE sheet.

In contrast to GC–MS, the microalga untreated LDPE sheet (Control) and *U. africanum* treated LDPE sheet (Sample) were taken for FT-IR analysis. The chemical bond includes C–H stretch (2915.76 cm^−1^ and 2847.97 cm^−1^), and bends (1462.69 cm^−1^ 1375.58 cm^−1^ and 729.35 cm^−1^) represents the presence of Alkanes (hydrocarbon) as a sole chemical functional group present in the control LDPE sheet (Fig. 15). Whereas, in microalga treated LDPE sheet, functional groups such as Carboxylic acids (O–H stretch at 3291.09 cm^−1^); Esters or saturated aliphatic (C=O stretch at 1745.03 cm^−1^); Alkenes (–C=C– stretch at 1646.53 cm^−1^); Nitro compounds (N–O asymmetric stretch at 1545.2 cm^−1^); Aliphatic amines (C–N stretch at 1017.32 cm^−1^) and Alkynes (–C≡C–H: C–H Bend at 667.31 cm^−1^) are present additional than the control LDPE sheet (Fig. 16). Therefore, the absence and presence of carboxylic acids, amino groups, nitro-compounds, and esters in control (microalga untreated LDPE sheet) and sample (*U. africanum* treated LDPE sheet) respectively, represents the biodegradable action of microalga on the LDPE sheet (Figs. 15, 16).

### Scanning electron microscopical (SEM) analysis of microalga treated LDPE sheet

**Figure 17:**
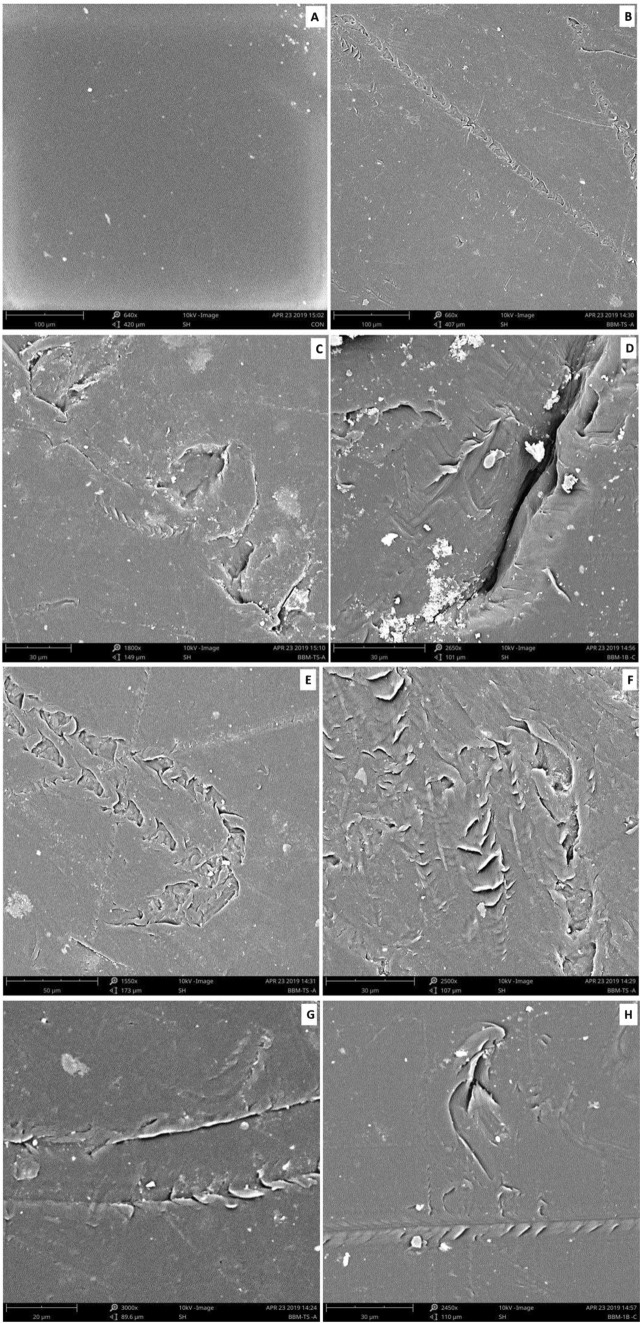
Scanning electron microscopical view of control and microalga treated LDPE sheet. (**A**) SEM image of untreated control LDPE sheet; (**B**–**H**) SEM images of *U. africanum* treated LDPE sheet showing erosions, abrasions, ridges and grooves on the surface of LDPE sheet.

The scanning electron microscopic images clearly reveals the presence of erosions, abrasions, grooves and ridges on the surface of the microalga treated LDPE sheet. Such kind of deteriorative actions were not seen in the microalga untreated control LDPE sheet (Fig. 17). Based on the high-resolution SEM imaging, it was very clear that the erosions, abrasions, grooves and ridges were formed by the colonization and degradation of LDPE by microalga *U. africanum*.

### Atomic force microscopical (AFM) analysis of microalga treated LDPE sheet

**Figure 18:**
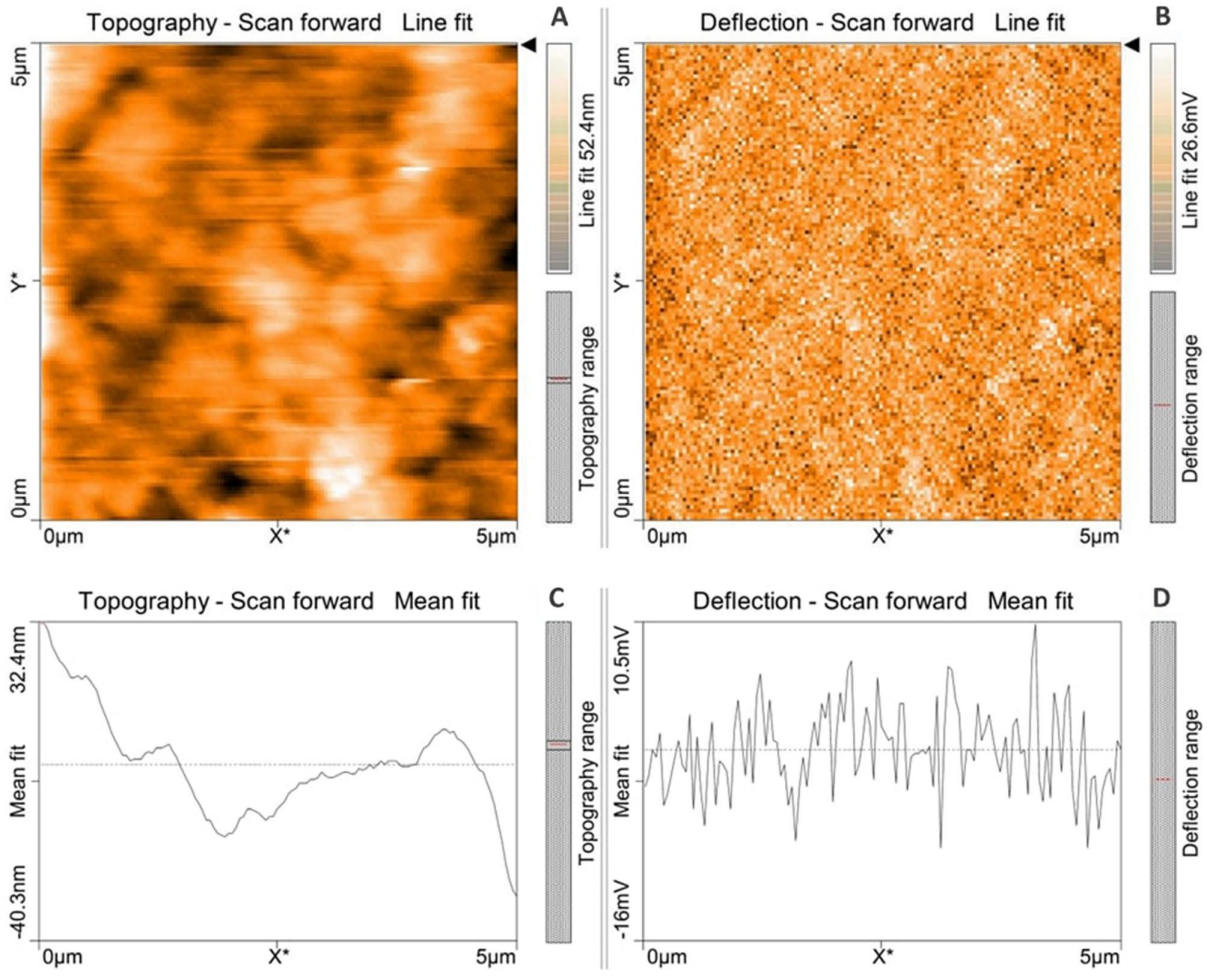
Atomic force microscopic results obtained for control (untreated) LDPE sheet of 5 µm^2^ area: (**A**) Topography of the surface (Line fit); (**B**) Voltage deflection (Line fit); (**C**) Topography with mean fit; and (**D**) Voltage deflection with mean fit.

**Figure 19:**
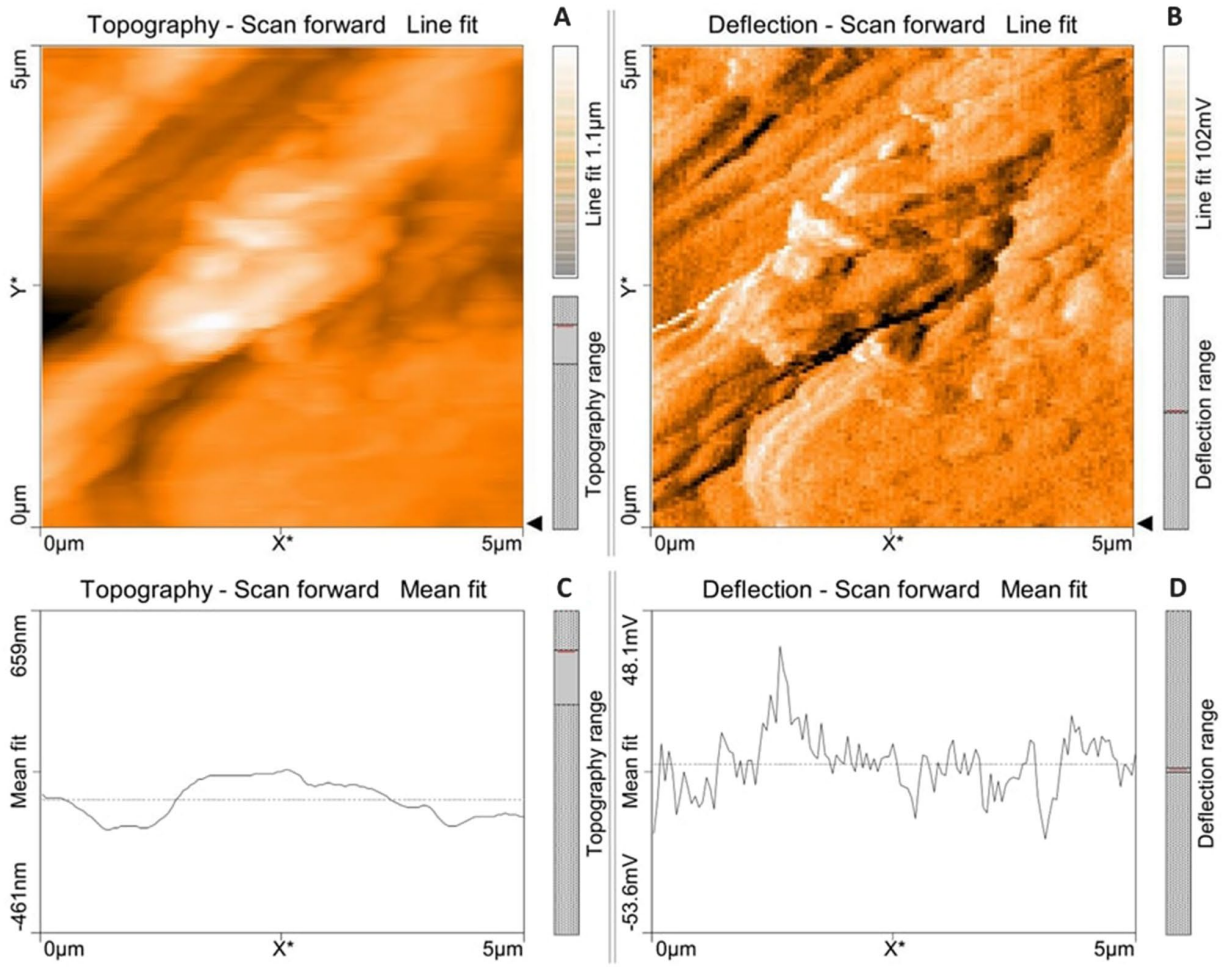
Atomic force microscopic results of microalga *U. africanum* treated LDPE sheet of 5 µm^2^ area: (**A**) Topography of the surface (Line fit); (**B**) Voltage deflection (Line fit); (**C**) Topography with mean fit; and (**D**) Voltage deflection with mean fit.

**Figure 20:**
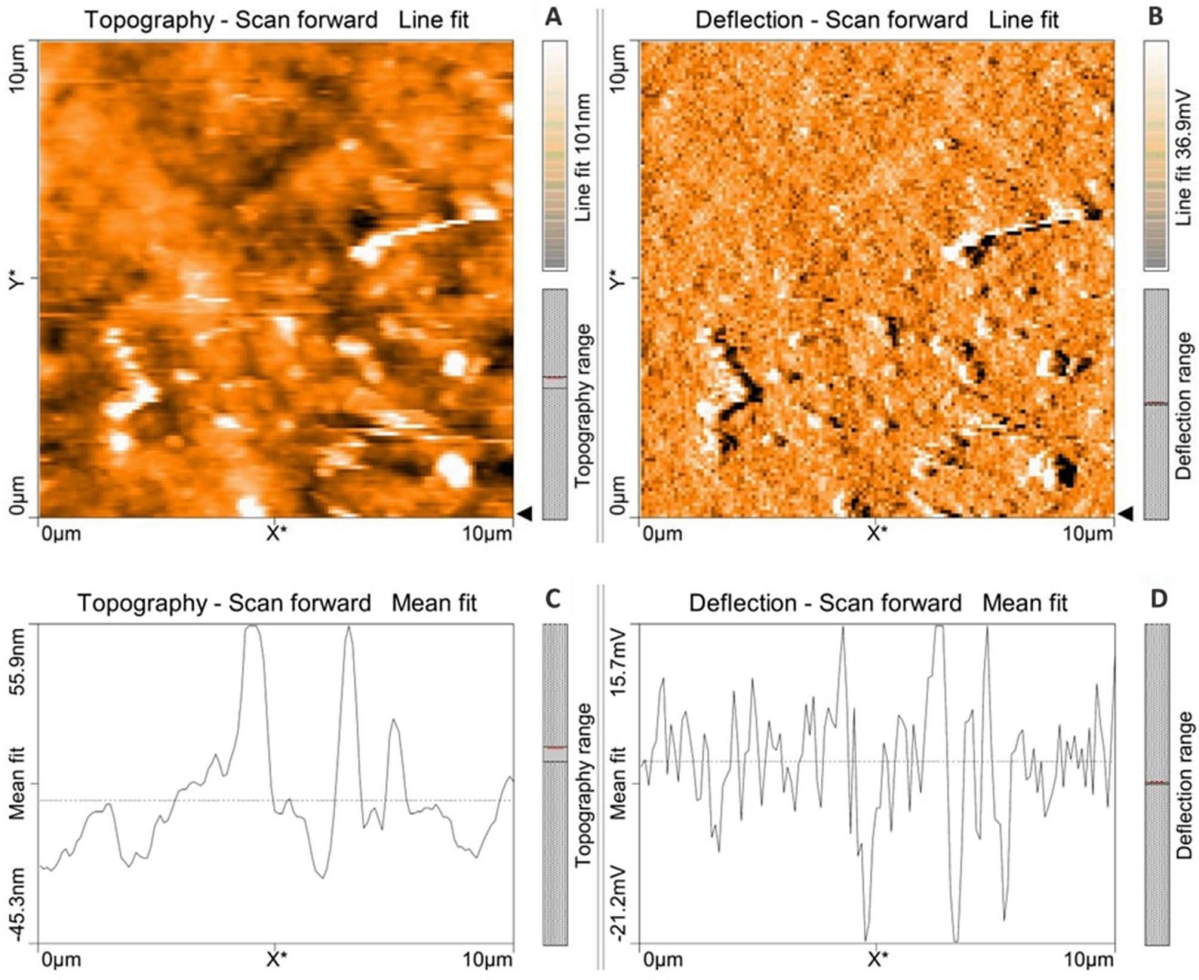
Atomic force microscopic results obtained for control (untreated) LDPE sheet of 10 µm^2^ area: (**A**) Topography of the surface (Line fit); (**B**) Voltage deflection (Line fit); (**C**) Topography with mean fit; and (**D**) Voltage deflection with mean fit.

**Figure 21:**
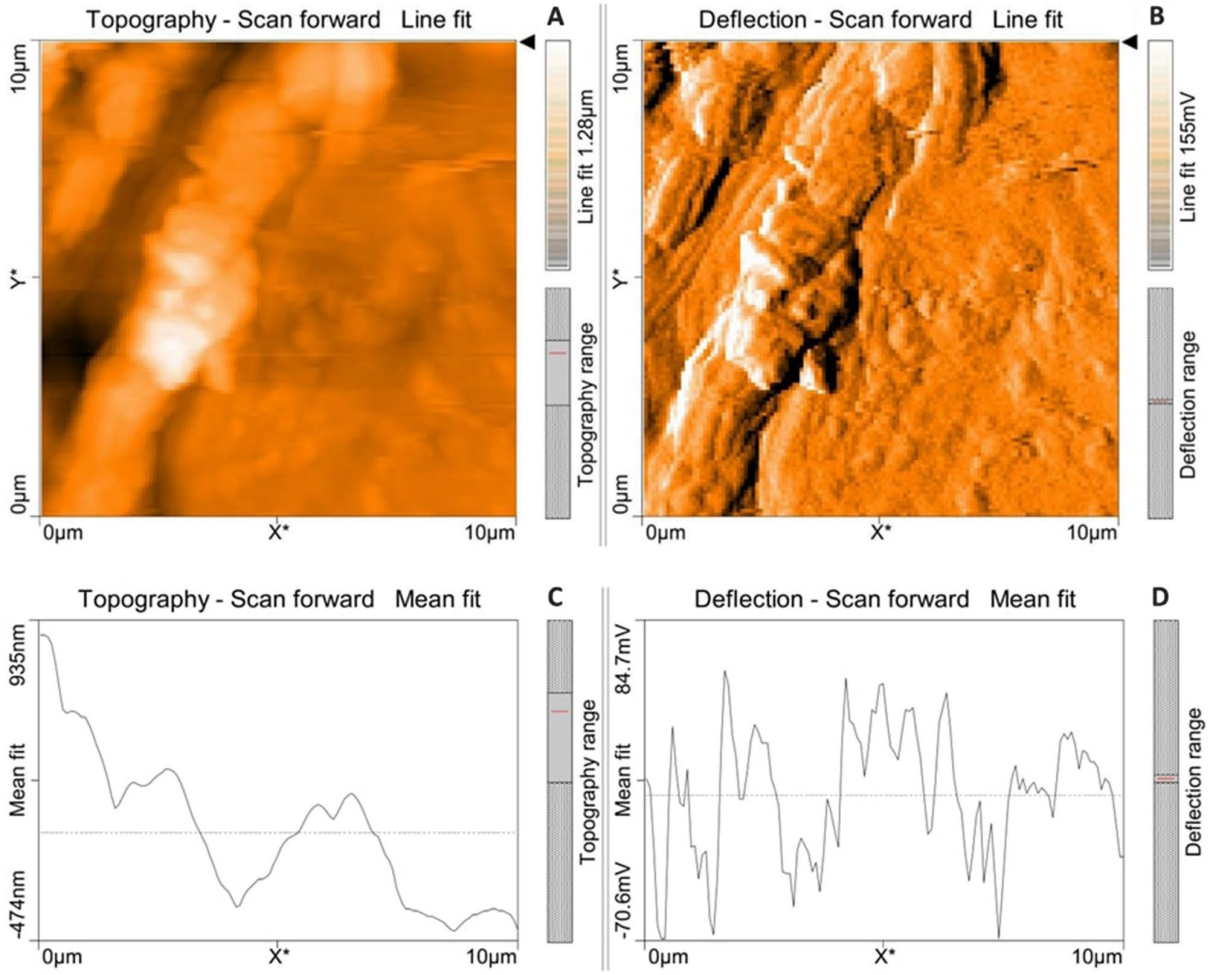
Atomic force microscopic results of microalga *U. africanum* treated LDPE sheet of 10 µm^2^ area: (**A**) Topography of the surface (Line fit); (**B**) Voltage deflection (Line fit); (**C**) Topography with mean fit; and (**D**) Voltage deflection with mean fit.

**Figure 22:**
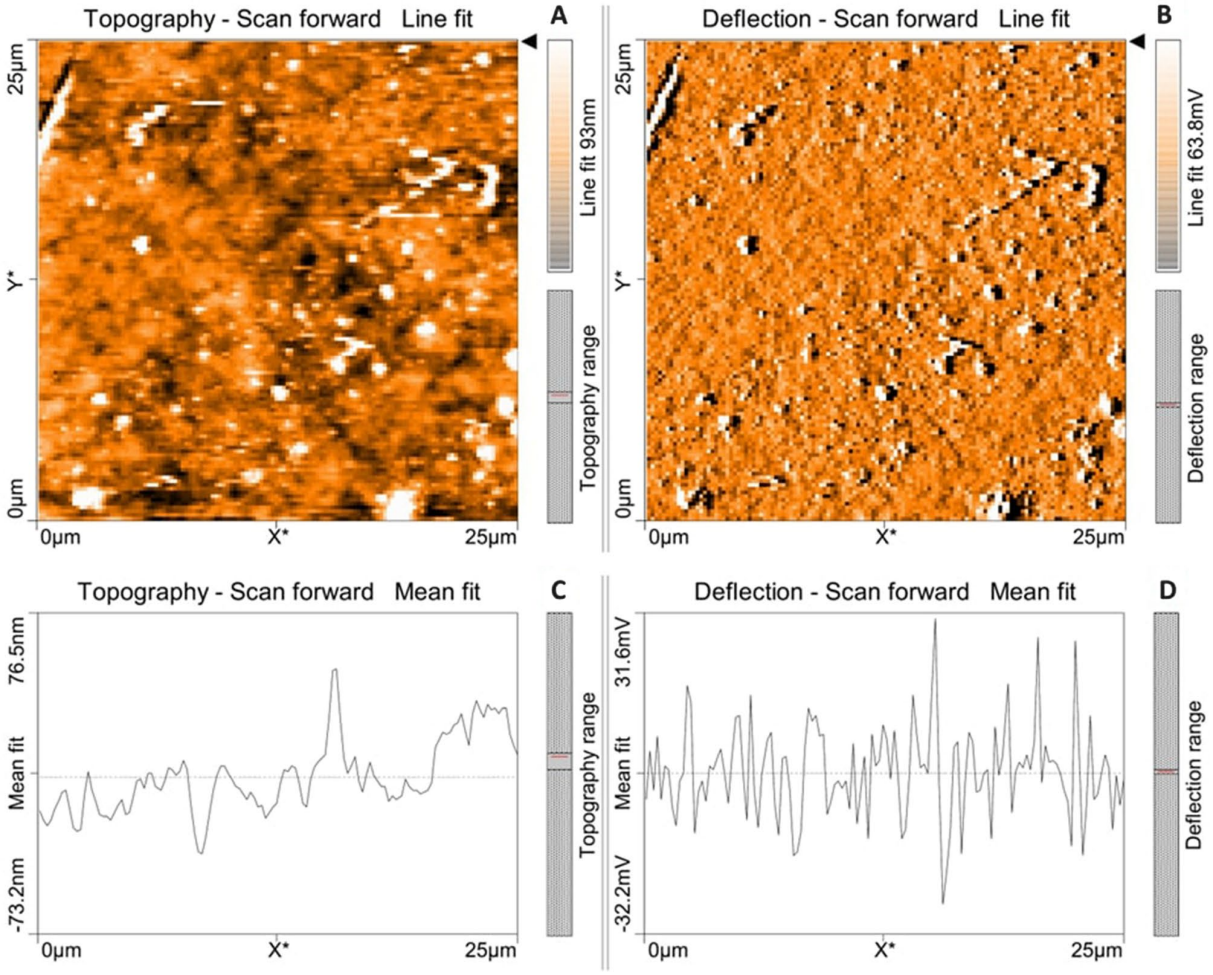
Atomic force microscopic results obtained for control (untreated) LDPE sheet of 25 µm^2^ area: (**A**) Topography of the surface (Line fit); (**B**) Voltage deflection (Line fit); (**C**) Topography with mean fit; and (**D**) Voltage deflection with mean fit.

**Figure 23:**
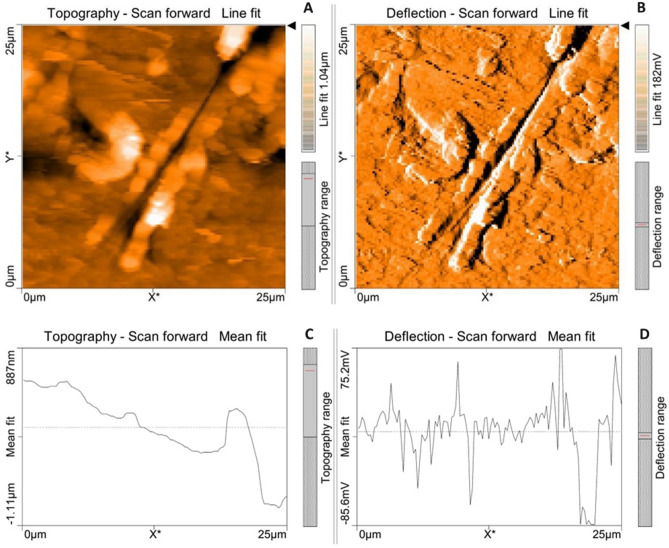
Atomic force microscopic results of microalga *U. africanum* treated LDPE sheet of 25 µm^2^ area: (**A**) Topography of the surface (Line fit); (**B**) Voltage deflection (Line fit); (**C**) Topography with mean fit; and (**D**) Voltage deflection with mean fit.

**Figure 24:**
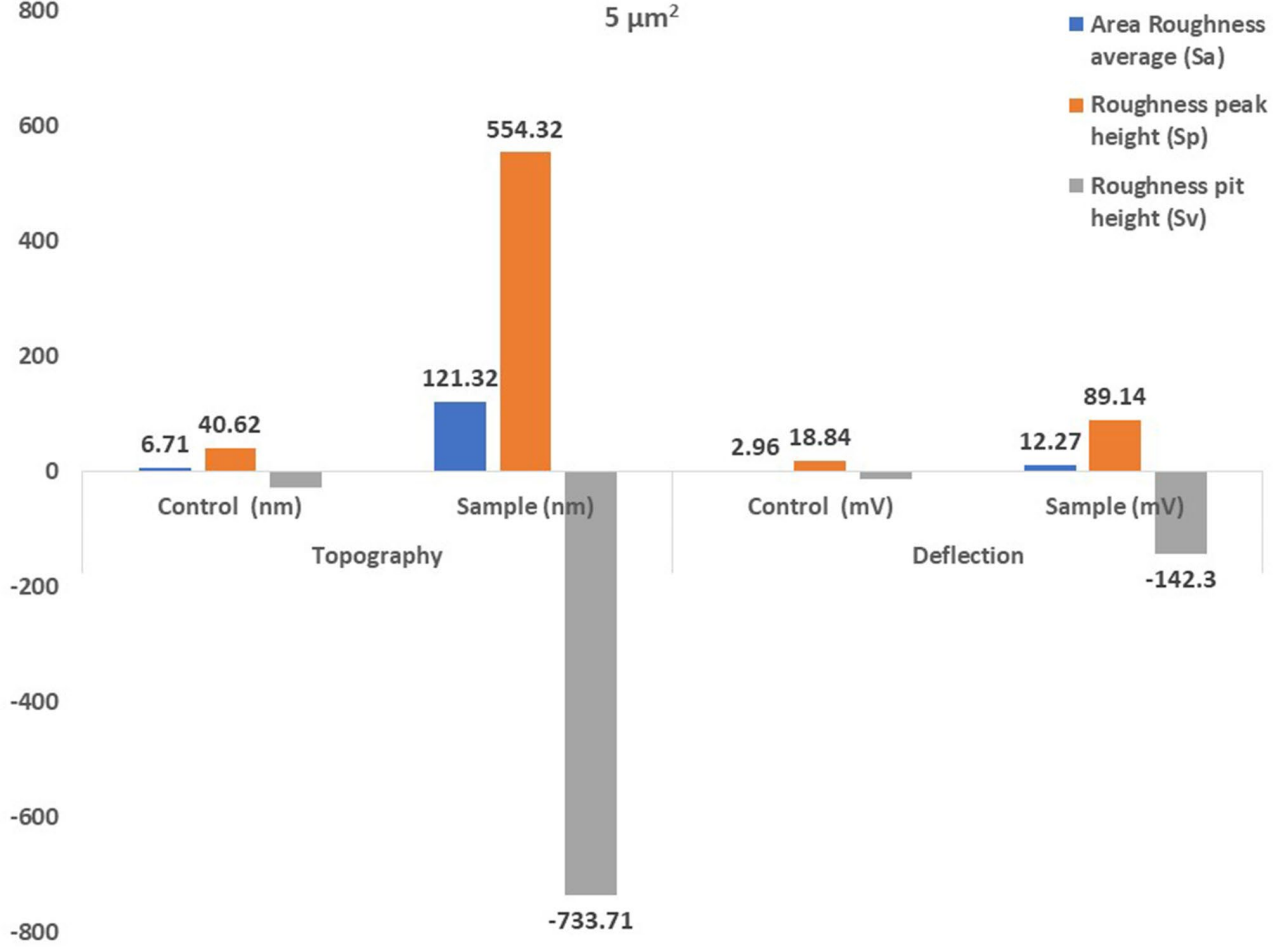
The bar graph showing differences in Area Roughness average (Sa), Roughness peak height (Sp), and Roughness pit height (Sv), comparatively between the control (untreated) and the *U. africanum* treated LDPE sheets of 5 µm^2^ area.

**Figure 25:**
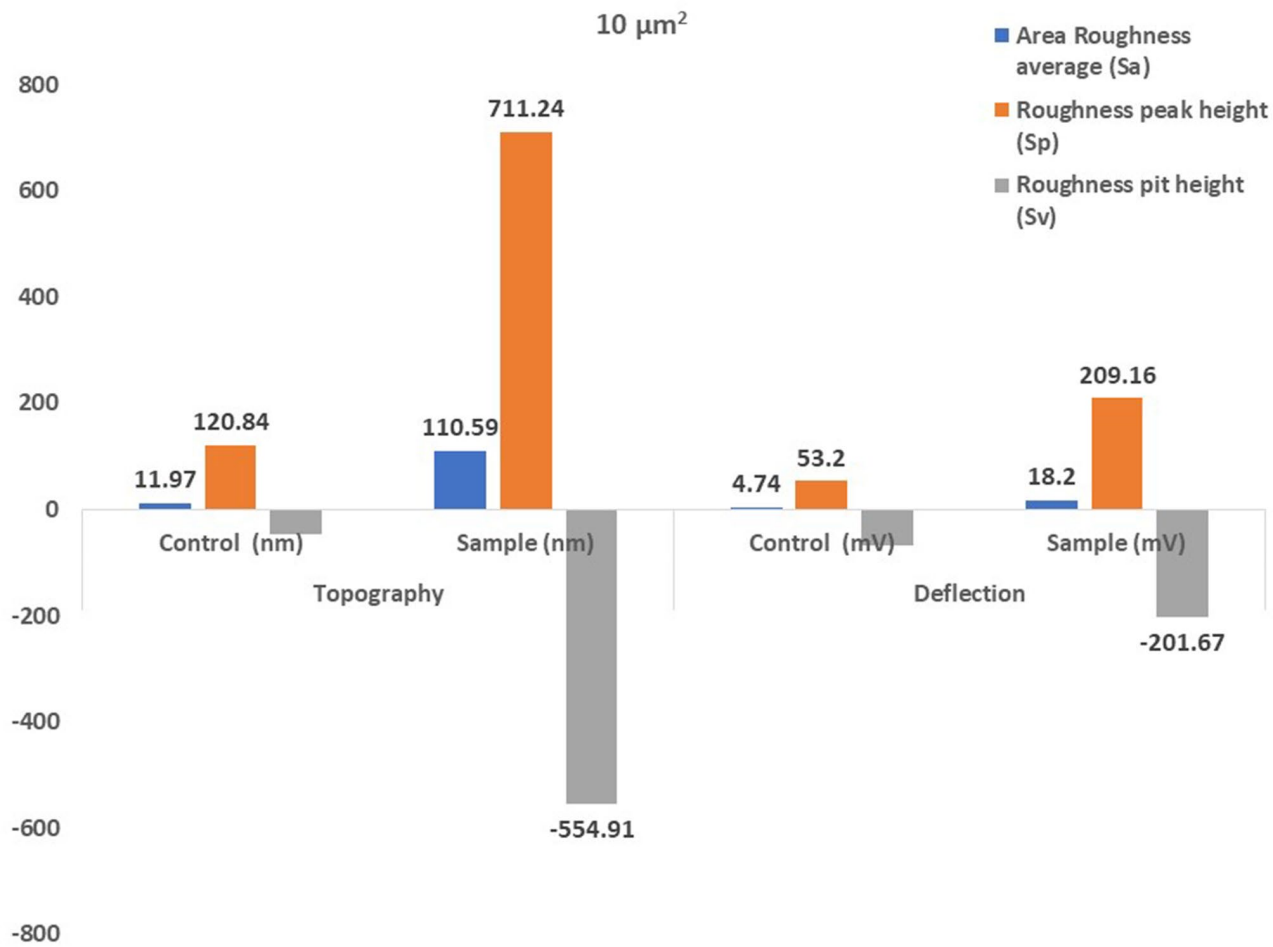
The bar graph showing differences in Area Roughness average (Sa), Roughness peak height (Sp), and Roughness pit height (Sv), comparatively between the control (untreated) and the *U. africanum* treated LDPE sheets of 10 µm^2^ area.

**Figure 26:**
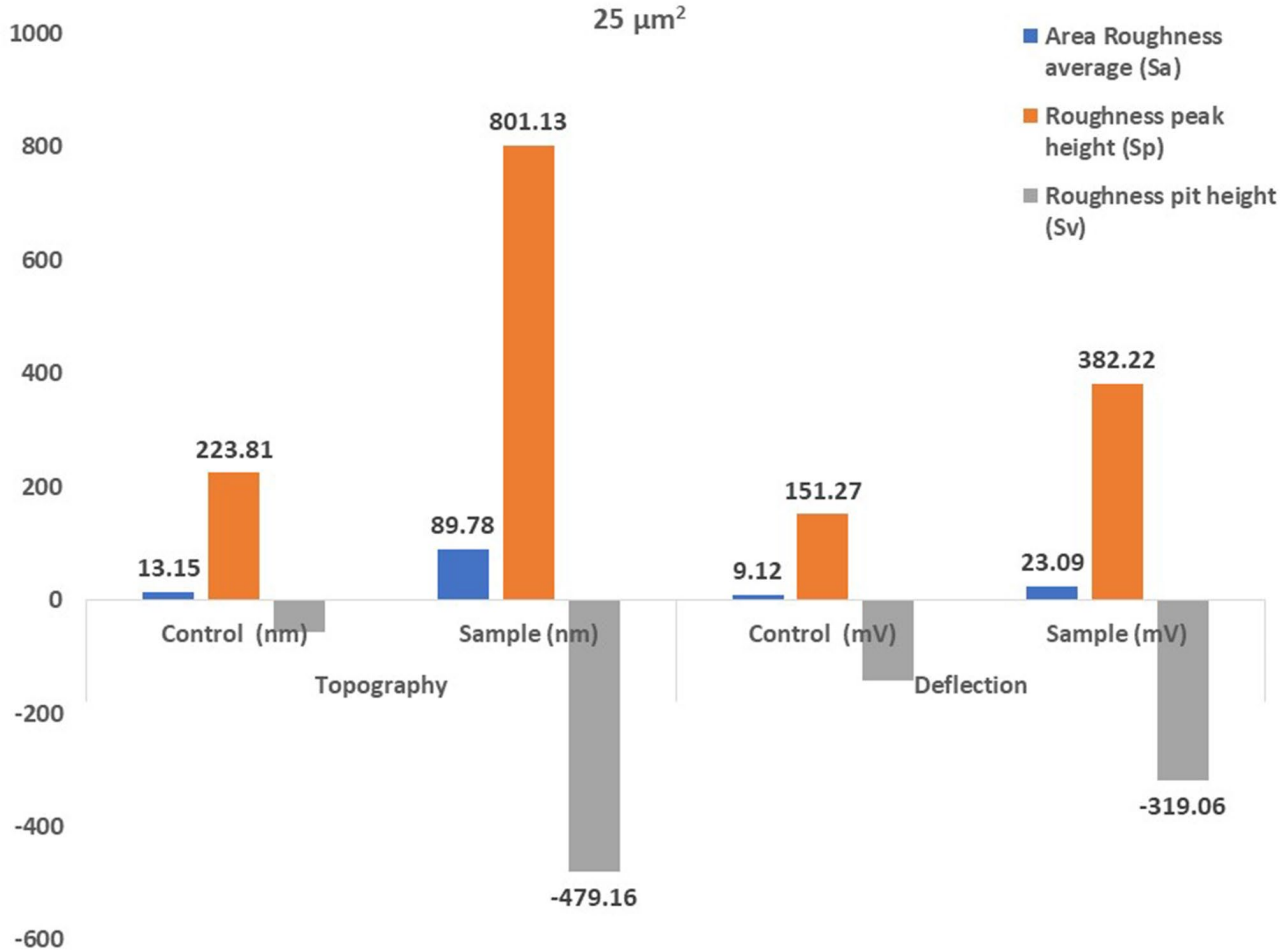
The bar graph showing differences in Area Roughness average (Sa), Roughness peak height (Sp), and Roughness pit height (Sv), comparatively between the control (untreated) and the *U. africanum* treated LDPE sheets of 25 µm^2^ area.

**Figure 27:**
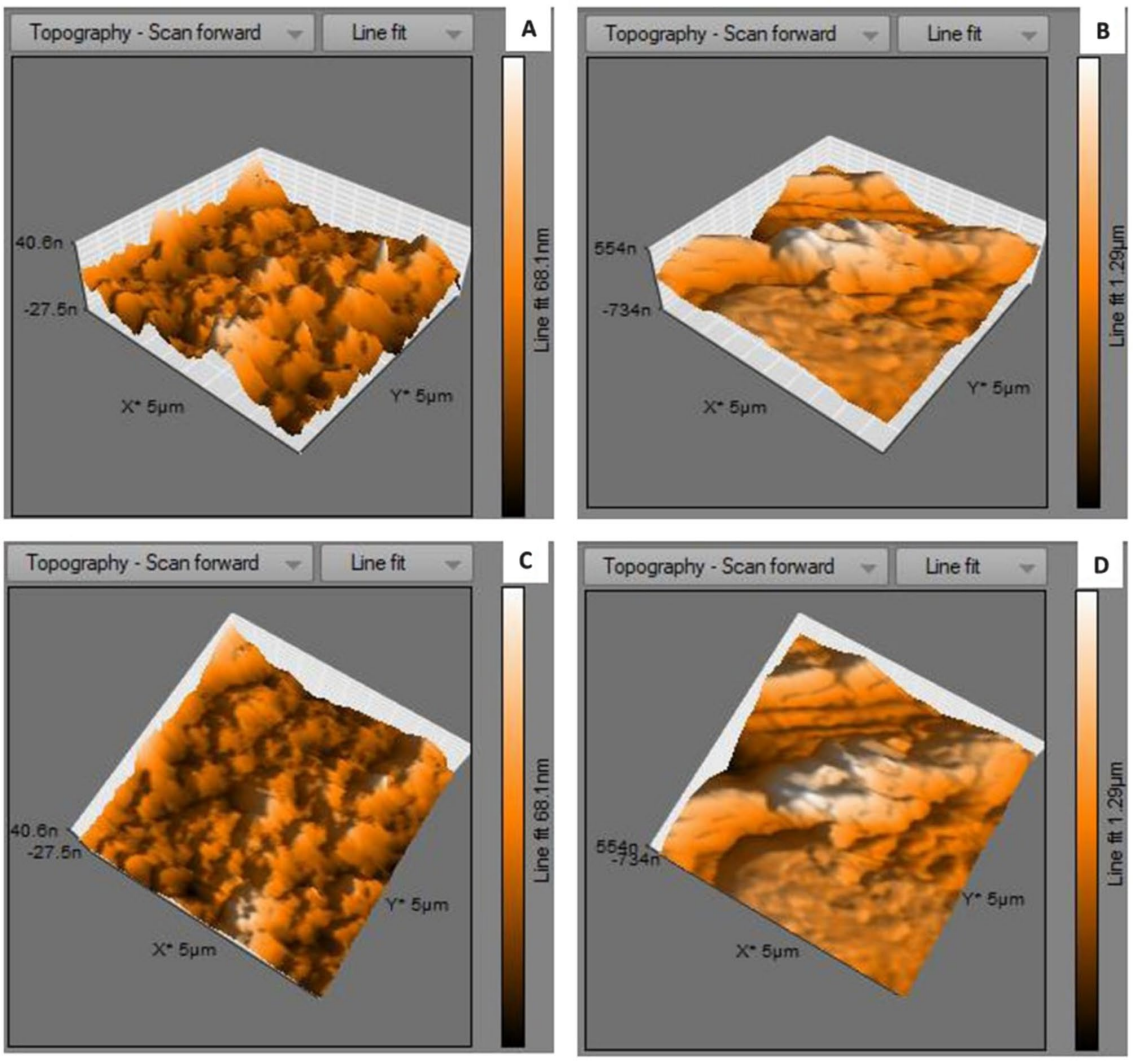
(**A**) Three-dimensional view of control (untreated) LDPE sheet of 5 µm^2^ area; (**B**) Three-dimensional view of *U. africanum* treated LDPE sheet; (**C**) Topographical view of control (untreated) LDPE sheet; (**D**) Topographical view of *U. africanum* treated LDPE sheet with erosions on its surface.

**Figure 28:**
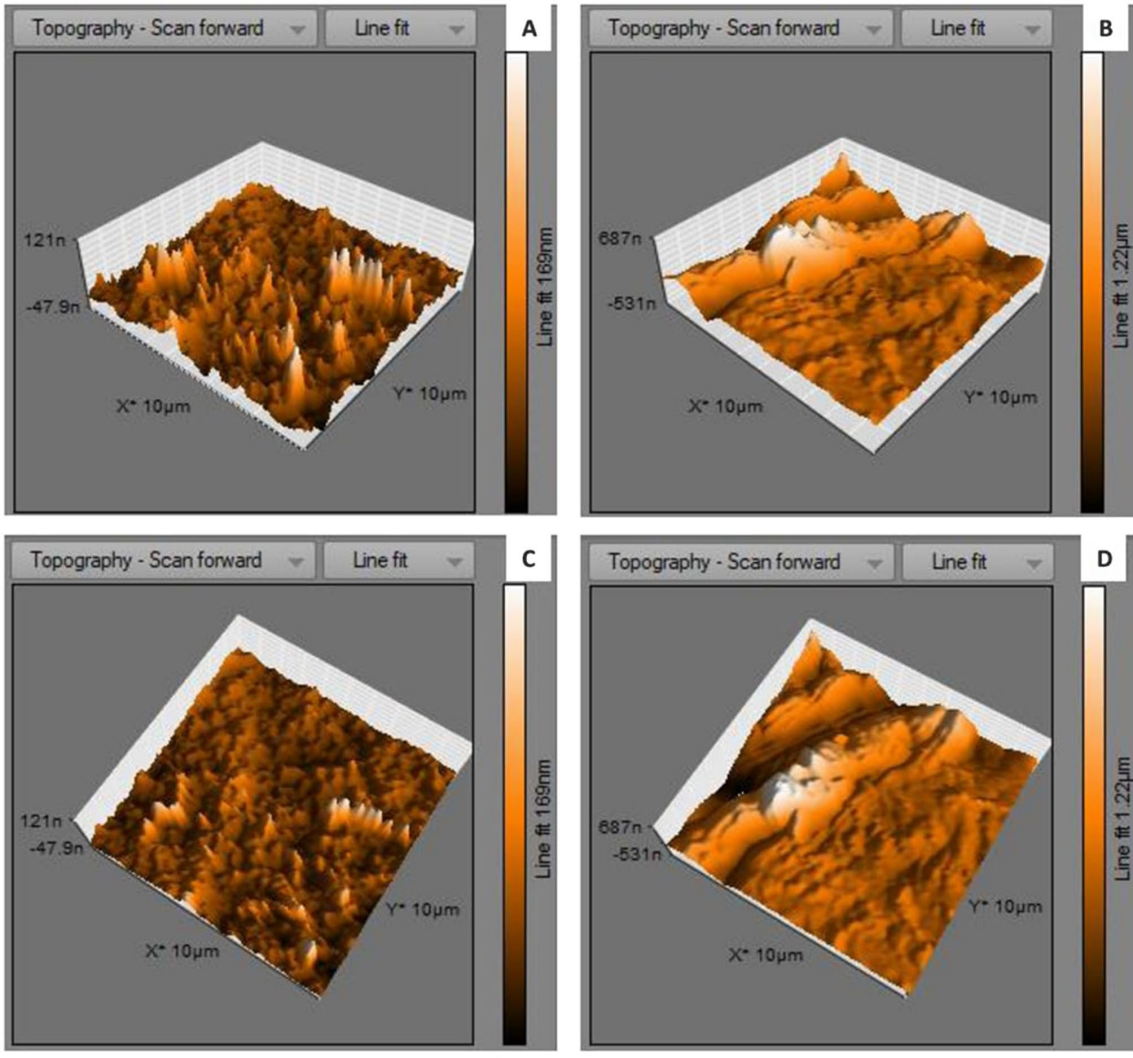
(**A**) Three-dimensional view of control (untreated) LDPE sheet of 10 µm^2^ area; (**B**) Three-dimensional view of *U. africanum* treated LDPE sheet; (**C**) Topographical view of control (untreated) LDPE sheet; (**D**) Topographical view of *U. africanum* treated LDPE sheet with erosions on its surface.

**Figure 29:**
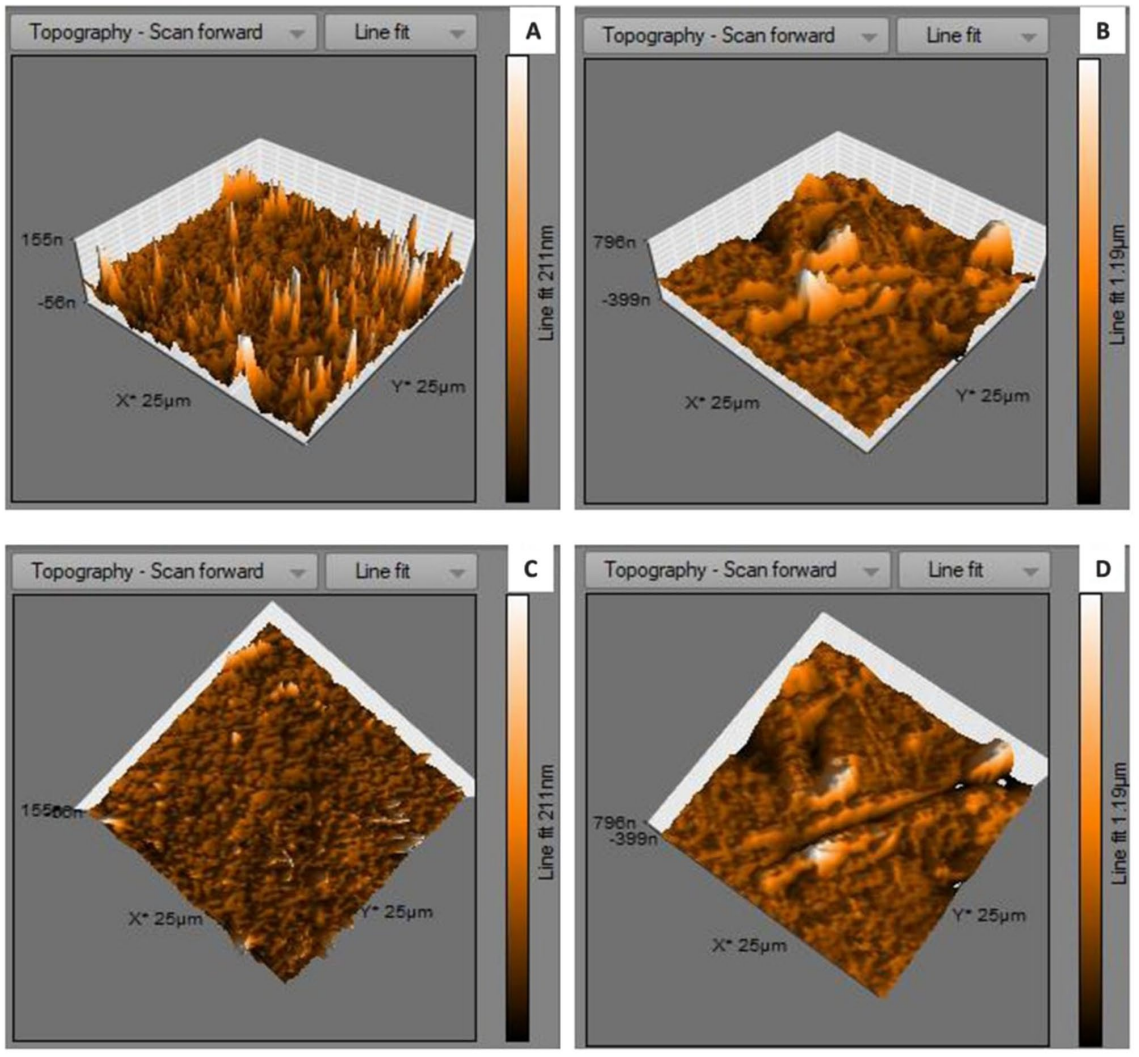
(**A**) Three-dimensional view of control (untreated) LDPE sheet of 25 µm^2^ area; (**B**) Three-dimensional view of *U. africanum* treated LDPE sheet; (**C**) Topographical view of control (untreated) LDPE sheet; (**D**) Topographical view of *U. africanum* LDPE sheet with erosions, grooves and ridges on its surface.

Atomic force microscopical analysis were carried out on the surface of both the control (microalga untreated LDPE sheet) and sample (*U. africanum* treated LDPE sheet) and resulted as 5 µm^2^ (Figs. 18, 19), 10 µm^2^ (Figs. 20, 21), and 25 µm^2^ (Figs. 22, 23) area squares. From which, the *U. africanum* treated LDPE sheet (sample) was found deteriorated, based on huge difference in the surface topography roughness and voltage deflection (Figs. 19, 21, 23). In this study, Area roughness average (Sa), Roughness peak height (Sp), and Roughness pit height (Sv) are the most important, and determining factor for the biodeterioration of LDPE sheet surface. Therefore, it very clear that there are huge differences in both the surface topography roughness and voltage deflection between the control (microalgae untreated LDPE sheet), and sample (*U. africanum* treated LDPE sheet). It is found that the Sa (Roughness average), Sp (Roughness peak height), and Sv (Roughness pit height) were higher in the *U. africanum* treated LDPE sheet (sample), than the microalga untreated LDPE sheet (control) in all the three 5 µm^2^ (Fig. 24), 10 µm^2^ (Fig. 25), 25 µm^2^ (Fig. 26) area squares. Similarly, *U. africanum* treated LDPE sheet was found eroded on the surface, and no such erosions were found on the surface of microalga untreated LDPE sheet in all the three 5 µm^2^ (Fig. 27), 10 µm^2^ (Fig. 28), 25 µm^2^ (Fig. 29) area squares.

**Figure 30:**
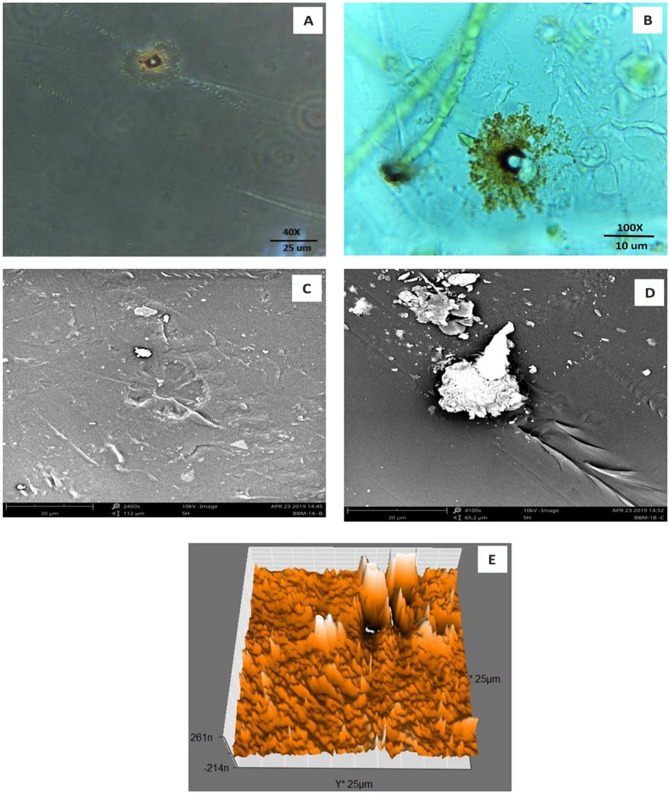
An eroded region on the surface of *Uronema africanum* treated LDPE sheet of different microscopical studies, such erosion was formed by the formation of the radial disc-like structure of the basal cell of the microalgal filament; (**A**) Dark field microscopy; (**B**) Light microscopy; (**C**) SEM image showing an impression caused by the formation the radial disc by the young germinating spore; (**D**) SEM image of an erosion caused by the formation of the disc by the microalga; (**E**) AFM image of the eroded region.

**Figure 31:**
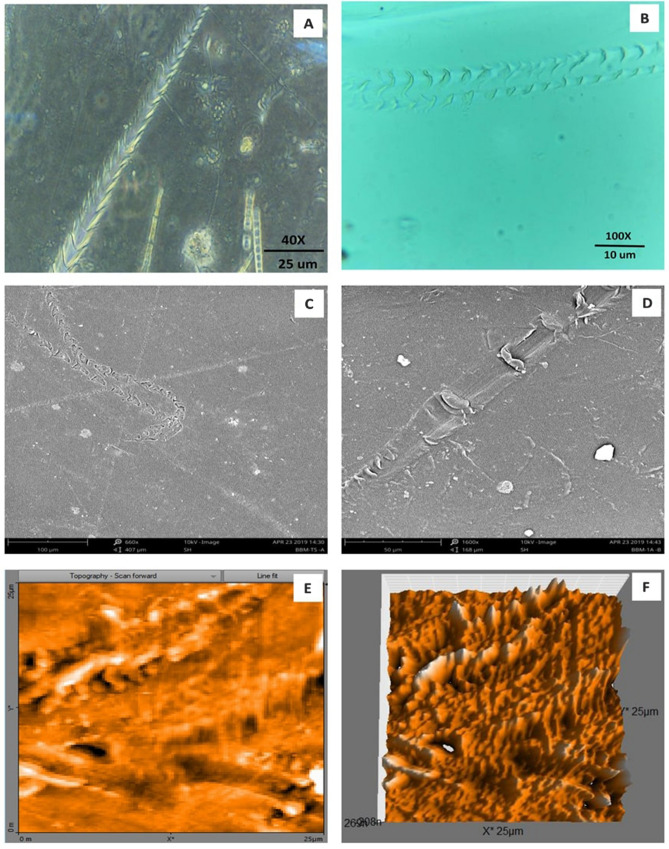
The images showing groove and ridges region on the surface of *Uronema africanum* treated LDPE sheet of different microscopical studies, such grooves and ridges were formed by the microalgal filaments; (**A**) Dark field microscopy; (**B**) Light microscopy; (**C**,**D**) SEM image of grooves and ridges formed by the microalga; (**E**) AFM—topography of the region where grooves and ridges were found; (**F**) AFM—topography—3D view of the region where grooves and ridges were found.

Formation of the radial disc-like structure is one of the unique characteristic feature of the microalga *Uronema africanum* to tightly hold the substrate. In our present study, the same radial disc-like structure found on the surface of microalga treated LDPE sheet which is optically seen by microscopical studies. Therefore, the overall microscopical studies resulted that, the dark field microscopy, optical microscopy, scanning electron microscopy and atomic force microscopy have shown the configuration of the radial disc-like structure were correspondingly equal in size at an average diameter of approximately 20–30 µm (Fig. 30). Similar kind of results were obtained for ridges and grooves also, which was configured by the microalgal filaments with an average size of 10–15 µm in width (Fig. 31).

## Discussion

The biodegradation of LDPE was reported in fungi, bacteria, and some actinomycetes (Streptomycetaceae). Very least works were reported on the biodegradation of LDPE by photosynthetic algae. Biodegradation of polyethylene by microalgae is an ecofriendly and cost-effectively viable option rather than conventional methods of degradation^22^. Commonly, *Oscillatoria, Phormidium, Lyngbya, Nostoc, Spirulina, Hydrocoleum, Chlorella, Pithophora, Stigeoclonium tenue, Anomoeoneis* and *Nitzschia* were reported to colonize the polyethylene bags in aquatic environment^31,32^. Since, the cyanobacteria are adhered strongly to the surface of polyethylene and were not removable by water jet^33^. Similarly, microalgal species includes 10 genera, 7 orders and 9 families were enumerated from the different polyethylene degrading sites of Kota, Rajasthan^24^. Whereas, in this present study, a green microalga, morphologically identified as *Uronema africanum* Borge was found colonized predominantly on the surface of the polyethylene bag collected from Kallukuttai Lake, near Taramani Railway Station, Chennai. The morphological features of the isolated microalga *U. africanum* was found similar with the morphological description given for *U. africanum* by Prasad and Misra^28,34^.

In our previous study, a cyanobacterium *Dolichospermum spiroides* was found to be colonized on the surface of the LDPE sheets^22^. But in this case, a green microalga *U. africanum* was responsible for the colonization of dumped LDPE sheets. Wherefore, such microalga was isolated to study the biological degradation of LDPE sheets. When the LDPE sheets were treated with *U. africanum* for 30 days of incubation, the LDPE sheets were completely colonized by the alga and green hair-like structures were seen nakedly protrude from the surface of the LDPE sheets. The *U. africanum* is a green filamentous alga, and hence the filaments were found attached perpendicular on the surface of LDPE sheet based on the light and dark-field microscopical studies. Concurrently, occurrence of erosions, abrasions, grooves and ridges on the treated LDPE sheets were clearly seen. And their configurations were optically corresponding to the radial disc attachment of the basal cell and filaments of the alga.

Gas chromatography and mass spectrometric analysis (GC–MS) and Fourier transform infra-red spectrometry (FT-IR) are the determining factor for biodegradation of polyethylene^35^. When polyethylene was biologically treated with *Klebsiella pneumoniae* CH001, generation of saturated fatty acids and carboxylic acids were confirmed by both GC–MS and FT-IR analysis respectively^36,37^. Similarly, most prominent structural changes were observed by FT-IR while LDPE degradation by a fungus *Aspergillus clavatus*^38^. In case of cyanobacteria, it may enhance the surface hydrophilicity of the polyethylene by forming additional carbonyl groups which can be further utilized by other microbes^39,40^. However, in our case, the GC–MS results clearly indicates the presence of hydrocarbons in the *U. africanum* treated sample (supernatant) when compared to control (microalga untreated supernatant). Simultaneously, from FT-IR results, functional groups such as carboxylic acids, esters, nitro compounds, and amino groups were determined in the sample (*U. africanum* treated LDPE sheet), but no such functional groups were found in the control (microalga untreated LDPE sheet). Therefore, our results clearly illustrate the biodeterioration of LDPE sheet by microalga *U. africanum* and found familiar with the results discussed by Vimala and Mathew^37^, and Awasthi et al.^36^.

Based on SEM analysis, microbes isolated from the forest soil and automobile wash-out sludge were found to degrade LDPE by forming cavities on its surface^41^. In our former study, three different microalgae including cyanobacteria, green alga, and diatom were involved to study the biodegradation of LDPE sheet. And among them, the cyanobacterium *Dolichospermum spiroides* alone forms a cavity on the surface of the treated LDPE sheet based on SEM analysis^22^. In another study, SEM images had shown profuse cracks on the LDPE surface by the adherence of the algae^21^. Similarly, by SEM analysis, erosion, pit formation, and cavities were clearly visible on the surface of the cyanobacteria (*Phormidium lucidum* and *Oscillatoria subbrevis*) treated LDPE sheets^33^. Simultaneously, in our study, the occurrence of erosions, abrasions, grooves, and ridges by the microalga *U. africanum* on the surface of LDPE sheets, were clearly visible by SEM analysis. Area roughness average (Sa), Roughness peak height (Sp), and Roughness pit height (Sv) were determined for both the *U. africanum* treated LDPE sheet (sample), and microalga untreated LDPE sheet (control) by AFM analysis. As a result, Sa, Sp, and Sv of both surface topography roughness, and voltage deflection were found higher in sample (*U. africanum* treated LDPE sheet) than the control (microalga untreated LDPE sheet). In adding to this, the configuration of abrasions found similar in size with the radial disc-like structure of the algae (20–30 µm). Simultaneously, the grooves and ridges also found similar to the filamentous nature of the algae (10–15 µm).

The biodegradation rate of polyethylene even after prolonged exposure up to 32 years in soil microbial consortia was found very low^42,43^. In another study, approximately 12 months incubation of LDPE sheet with microbes increase surface roughness when compared with the control, causing deterioration. *Aspergillus clavatus* treated LDPE sheet for 90 days of incubation in aqueous medium, had shown fractures, erosion and grooves without any prior treatment of the LDPE sheet^38^. Whereas, 126 days were taken for forming small cavities, and pits on the surface of LDPE sheet by the fungal mycelium of *A. niger*^44^. Weight loss of thermally treated HDPE and LDPE sheets were observed about 9% and 19% respectively, but it was 3.5% and 10% in the case of bacteria, *Bacillus sphaericus* treated samples for a period of 12 months^45^.

Awasthi et al.^8^ exemplified that the biodegradation of polyethylene by *Klebsiella pneumoniae* CH001 took only 60 days. Whereas, in the case of fungi *Aspergillus niger* and *A. clavatus*, it was 126 days and 90 days respectively^38,44^. *Azotobacter* sp. reported to degrade polyethylene within 45 days of incubation^41^. Cyanobacteria, *Phormidium lucidum* and *Oscillatoria subbrevis* have been reported to degrade polyethylene within 6 weeks of incubation^33^. Based on our present study, the microalga *Uronema africanum* initiated polyethylene (LDPE) degradation within 30 days of incubation.

Therefore, based on the results obtained from our study, this is a *hitherto* report on the biodegradation of LDPE sheet by photosynthetic, and filamentous microalga *Uronema africanum* Borge.

## Materials and methods

### Collection of samples

Dumped waste carry bag sample and water sample were collected from the Kallukuttai lake, nearby Taramani Railway Station, Chennai, Tamil Nadu, India on 28th December 2018. The samples were collected in a sterile polyethylene bags and brought to the laboratory immediately for further processes.

### Isolation of microalga colonized on LDPE sheet

For isolation of microalga colonizing on the surface of LDPE sheet, we have used three different culture media Bold basal medium (BBM)^46^, BG 11^47–49^ and DM (Diatom medium)^50,51^ to target Chlorophyceae (Green algae), Cyanophyceae (Blue-green algae) and Bacillariophyceae (Diatoms) members respectively.

About 250 ml conical flasks were used with 100 ml of culture medium followed by supplementation of 10 numbers of LDPE (low density polyethylene) sheets of 1 cm^2^. The LDPE sheets were supplemented after autoclaving of the culture medium at 15 psi for 15 min. About an inoculation loop full of samples from the collected waste carry bags were inoculated and incubated under light illumination for 12:12 h of light and dark conditions at algal culture room (25 °C). The most common microalga colonizing the LDPE sheet was isolated, sub-cultured and maintained in the algal culture room based on streak plate method.

### Morphological identification of selected microalga

Morphological characteristic features of the selected microalga were observed and studied microscopically. Based on the morphology, the microalga was identified with the help of Dr. B. Babu, Assistant Professor, Department of Plant Biology and Plant Biotechnology, Madras Christian College (Autonomous), Chennai, 600059. Meanwhile, the accepted taxonomic name of the microalga was confirmed by database ‘algaebase’ (http://www.algaebase.org)^30^.

### Biodegradation of LDPE sheet by selected microalga

The microalga was selected based on the colonization on the surface of the LDPE sheet. Furthermore, the selected and isolated pure microalga was subjected to treat LDPE sheets for its biodegradation studies. In an autoclaved 100 ml of BBM culture medium in a 250 ml conical flask, 10 numbers of 1 cm^2^ LDPE sheets were added and the pure selected microalga was inoculated (1% inoculum) and incubated under 12:12 h of light and dark condition in an algal culture room. After 30 days of incubation, the LDPE sheets were taken out from the culture flasks and analyzed for its biodegradation by microalga.

### Optical microscopy on biodegradation of LDPE sheet

For optical microscopical study on biodegradation of LDPE sheet by the selected microalga; sophisticated, binocular, compound microscope was employed (Lawrence and Mayo). The image was focused at 10 ×, 40 × and 100 × (oil immersion) magnification and captured using an inbuilt camera along with the microscope using Scopeimage 9.0 software.

### Dark field microscopy on biodegradation of LDPE sheet

The biodegradation of LDPE sheet by the selected microalga was further studied and confirmed by dark field microscopy (CETI). The images were focused at 10 ×, 20 × and 40 × and captured by the inbuilt camera with the microscope using Scopeimage 9.0 software. Dark field images were captured for confirmation of biodegradation of LDPE sheet by the microalga.

### Gas chromatography and mass spectrometric analysis of control and microalga treated LDPE sheet sample

After microalgal treatment of LDPE sheet, the culture medium was centrifuged at 5000 rpm for 10 min. and the supernatant was evaporated and extracted with methanol, followed by GC–MS analysis. Microalgal culture without LDPE treatment was used as a negative control. The gas chromatography was performed by using Perkin Elmer Clarus 680 instrument equipped with fused silica column with Elite-5MS (30 m × 0.25 mm ID × 250 µm df). The mass spectrometry was done by using Perkin Elmer Clarus 600 (EI), the conditions were 240 °C for transfer line temperature the same temperature was followed as ion source temperature. The contents were determined by GC–MS NIST (2008).

### Fourier transform infrared spectroscopy (FT-IR) analysis of microalga treated LDPE sheet

The microalga treated LDPE sheets were analyzed by Infra-Red spectrometer (Bruker, INVENIO R). The FT-IR spectrum of microalga treated LDPE sheet was obtained as a percentage of transmission ranged from 400 to 4000 cm^−1^. The results of microalga treated LDPE sheet was compared with the results obtained from the untreated LDPE sheet (control).

### Scanning electron microscopical (SEM) analysis of microalga treated LDPE sheet

The microalga treated LDPE sheet was subjected to analyze under scanning electron microscopy (SEM). Prior to SEM analysis, the sample could ionize by ion sputter on a metal stub for 20 min. After gold coating, the samples were kept under vacuum to view microscopically by a desktop scanning electron microscope (SEM) (Phenom World Pro) and photographed. For control sample, a fresh and untreated LDPE sheet was employed.

### Atomic force microscopical (AFM) analysis of microalga treated LDPE sheet

To study the surface erosion and biodegradation of LDPE sheet by biological treatment of the selected microalga, atomic force microscopy was employed. Atomic scope microscope Nanosurf Easyscan 2, head type EZ2-AFM with scan head 10-06-176.hed was used. Cantilever type: ContAI-G was used with static force as operating mode and Air was the measurement environment. The Z-controller was set at setpoint: 20 nV, with I-gain: 1000 and P-gain: 10,000. The AFM was controlled using the software version 3.0.1.16 and firmware version 3.1.3.0. The images were taken at 25 µm^2^, 10 µm^2^ and 5 µm^2^ sizes and analyzed with different mode by software Nanosurf easyscan 2 version 3.8.6.3. A fresh untreated LDPE sheet was used as control.

## Conclusion

In our study, we have isolated a microalga *Uronema africanum* Borge found colonized on the surface of dumped waste plastic carry bags from highly urbanized freshwater lake. The isolated microalga was subjected to biodegradation of LDPE sheets. Based on the light microscopy, dark field microscopy, GC–MS, FT-IR, SEM and AFM results, it was found that the microalga can initiate degradation of low-density polyethylene in 30 days of incubation. Intriguingly, the configuration of the radial disc-like attachment structure of the microalga was found corresponding to the abrasions on the surface of LDPE sheet at an average size of 20–30 µm in diameter. Whereas, the configuration of ridges and grooves were found similar with the filamentous nature of the microalga (10–15 µm width). Therefore, this is a *hitherto* report on the biodegradation of LDPE sheet by the microalga *Uronema africanum* Borge.
